# Substratum stiffness regulates Erk signaling dynamics through receptor-level control

**DOI:** 10.1016/j.celrep.2021.110181

**Published:** 2021-12-28

**Authors:** Payam E. Farahani, Sandra B. Lemke, Elliot Dine, Giselle Uribe, Jared E. Toettcher, Celeste M. Nelson

**Affiliations:** 1 Department of Chemical & Biological Engineering, Princeton University, Princeton, NJ 08544, USA; 2 Department of Molecular Biology, Princeton University, Princeton, NJ 08544, USA; 3 Lead contact

## Abstract

The EGFR/Erk pathway is triggered by extracellular ligand stimulation, leading to stimulus-dependent dynamics of pathway activity. Although mechanical properties of the microenvironment also affect Erk activity, their effects on Erk signaling dynamics are poorly understood. Here, we characterize how the stiffness of the underlying substratum affects Erk signaling dynamics in mammary epithelial cells. We find that soft microenvironments attenuate Erk signaling, both at steady state and in response to epidermal growth factor (EGF) stimulation. Optogenetic manipulation at multiple signaling nodes reveals that intracellular signal transmission is largely unaffected by substratum stiffness. Instead, we find that soft microenvironments decrease EGF receptor (EGFR) expression and alter the amount and spatial distribution of EGF binding at cell membranes. Our data demonstrate that the mechanical microenvironment tunes Erk signaling dynamics via receptor-ligand interactions, underscoring how multiple microenvironmental signals are jointly processed through a highly conserved pathway that regulates tissue development, homeostasis, and disease progression.

## In brief

Farahani et al. investigate how soft microenvironments limit signal transmission through the EGFR/Erk pathway. Optogenetic stimulation reveals that the intracellular portion of the EGFR/Erk pathway remains signaling competent regardless of substratum stiffness. Rather, soft microenvironments limit Erk signaling by decreasing interactions between EGFR and its cognate ligand.

## Graphical Abstract

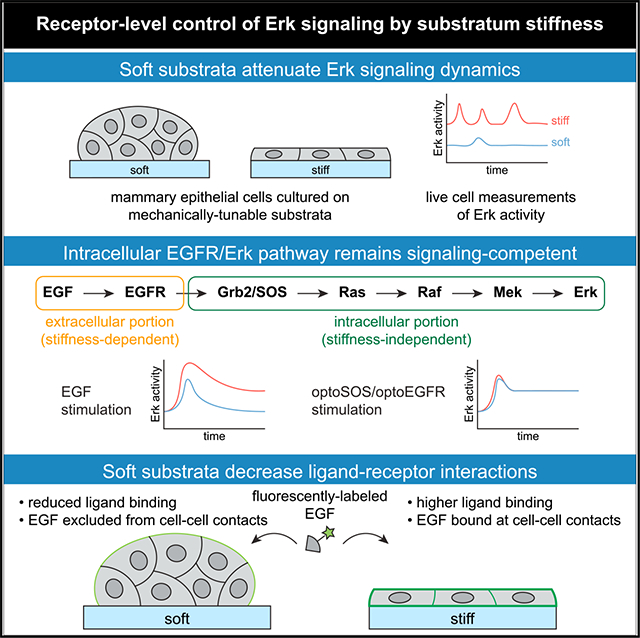

## INTRODUCTION

In growth factor signaling, extracellular ligands bind to receptor tyrosine kinases at the cell surface, initiating a cascade of signaling events to phosphorylate the terminal kinase Erk and trigger downstream cellular processes including growth, proliferation, and migration. The dynamics of Erk activation have long been hypothesized to play a crucial role in determining which cellular response programs are initiated. Different oncogenic mutations, growth factor receptors, and even ligands for the same receptor can shift Erk activity from transient (<30 min) pulses to sustained (hours-long) signaling, changes that are thought to select distinct response programs (Bishop et al., 1994; Marshall, 1995). Indeed, recent experiments that directly perturb Erk dynamics have demonstrated that altered dynamics can switch cell fates during embryogenesis (Johnson and Toettcher, 2019) and drive improper gene expression and proliferation in tumor cells (Bugaj et al., 2018). The advent of live-cell Erk biosensors has uncovered ornate pulsatile Erk dynamics in cells (Albeck et al., 2013; Aoki et al., 2013) and traveling waves across tissues (Hiratsuka et al., 2015), suggesting that a full accounting of dynamics-influenced cell behaviors remains incomplete.

Most efforts to understand Erk dynamics have focused on how they are altered by extracellular ligands (Albeck et al., 2013; Freed et al., 2017), growth factor receptors (Kiyatkin et al., 2020; Santos et al., 2007), oncogene expression (Aikin et al., 2020; Bugaj et al., 2018), and pharmacological agents (Gerosa et al., 2020; Goglia et al., 2020). However, the mechanical properties of the microenvironment play a critical role in regulating Erk and downstream processes such as tissue homeostasis and tumor progression (Mohammadi and Sahai, 2018). For instance, exposure to stiff microenvironments induces malignant cell behavior by increasing signaling through the Erk pathway (Paszek et al., 2005). Yet how the mechanical microenvironment influences Erk signaling dynamics remains poorly characterized. Early studies in fibroblasts implicated virtually every signaling node in the Erk pathway as a target of crosstalk from integrin engagement, including the activation of multiple growth factor receptors (Giancotti and Ruoslahti, 1999), Ras (DeMali et al., 1999), Raf (Lin et al., 1996), Mek (Renshaw et al., 1997), and the nuclear translocation of Erk (Aplin et al., 2001). Subsequent studies employing synthetic substrata to mimic the mechanical properties of native tissues determined that soft microenvironments decrease population-averaged levels of Erk phosphorylation in multiple cell types, including mammary epithelial cells (Paszek et al., 2005), kidney epithelial cells (Kim and Asthagiri, 2011), and epidermal stem cells (Trappmann et al., 2012). Most recently, live-cell studies have shown that Erk activity is regulated by cell density (Aoki et al., 2013), integrin expression (Hiratsuka et al., 2020), and protrusive forces (Yang et al., 2018). Despite these advances, it is still unknown how single-cell Erk dynamics vary as a function of substratum stiffness and which mechanically regulated steps control the response of growth factor signaling through Erk.

Here, we set out to define the relationship between substratum stiffness and Erk signaling in the MCF10A human mammary epithelial cell line, a canonical model system for both mechano-transduction (Aragona et al., 2013; Paszek et al., 2005) and Erk signaling dynamics (Aikin et al., 2020; Albeck et al., 2013). We monitored steady-state and EGF-stimulated Erk dynamics in cells cultured on substrata with stiffnesses ranging from those of the soft, normal mammary gland (0.1 kPa) to stiff mammary tumors (4 kPa). These experiments revealed several stiffness-dependent changes in Erk signaling dynamics, including a decrease in the amplitude and frequency of Erk activity pulses and a shift from transient to sustained growth factor-stimulated Erk signaling. We then used optogenetic stimuli at different signaling nodes to pinpoint mechanically regulated steps in the Erk pathway. Optogenetic stimulation of Ras or EGFR drove similar responses regardless of substratum stiffness, revealing the intracellular Erk pathway to be relatively insensitive to changes in the mechanical microenvironment. In contrast, we found that soft microenvironments inhibit EGFR-level signaling through multiple mechanisms: by downregulating receptor expression and altering the amount and pattern of EGF binding at the cell surface. Ectopically increasing EGFR expression in cells in soft microenvironments was sufficient to increase EGF membrane binding and internalization, and shift growth factor-dependent Erk signaling to levels found in stiff microenvironments. Taken together, our findings reveal how substratum stiffness tunes signal transmission along the EGFR/Erk pathway, implicating ligand-receptor interactions as a key signaling step accentuated by stiff microenvironments.

## RESULTS

### Soft substrata attenuate the dynamics of Erk signaling

**Figure 1.**
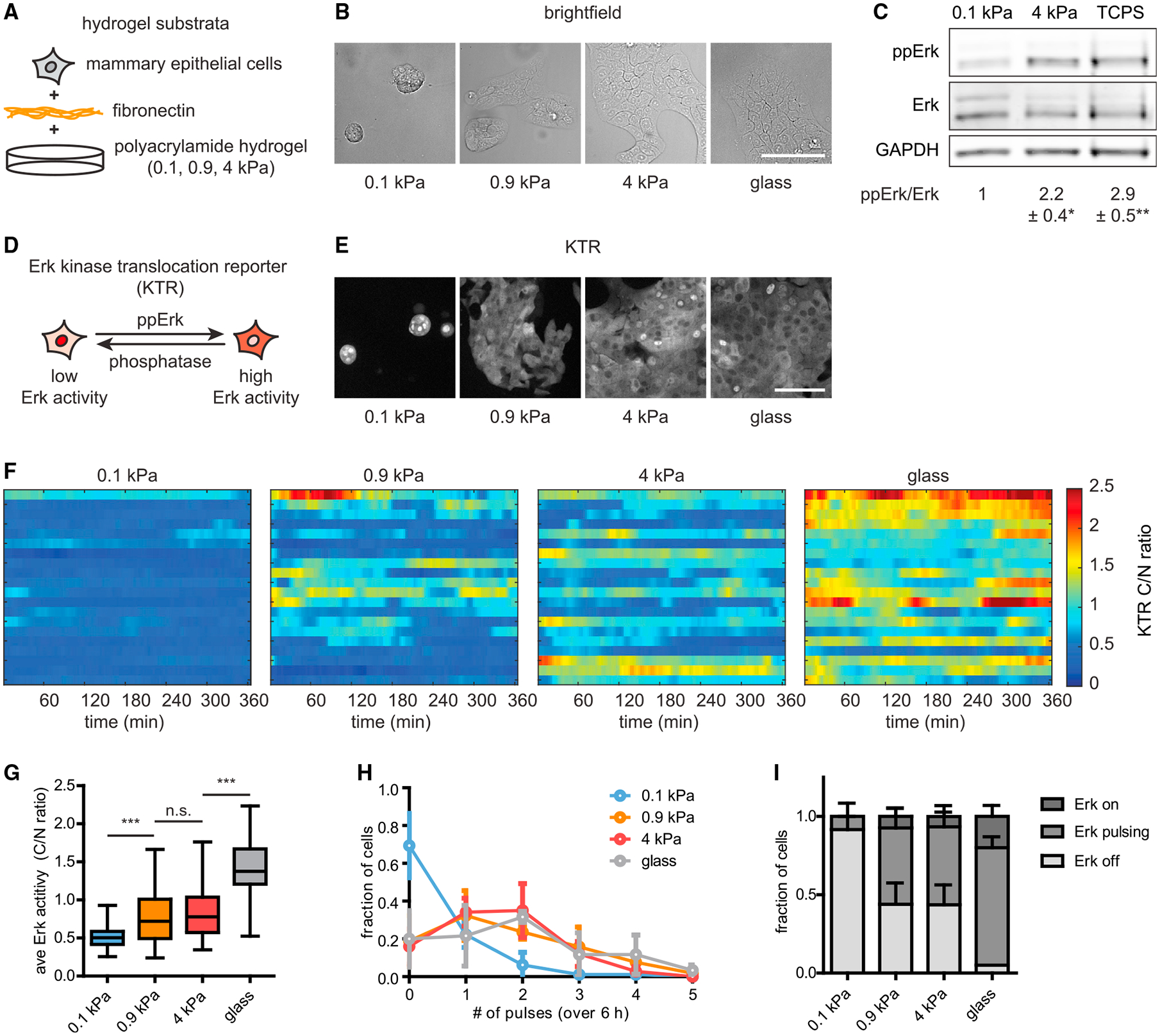
Substratum stiffness regulates Erk signaling dynamics (A) MCF10A human mammary epithelial cells were cultured on fibronectin-coated polyacrylamide hydrogels, the elastic moduli of which were tuned to mimic stiffnesses ranging from those of the soft, normal mammary gland to stiff mammary tumors. (B) Brightfield images of MCF10A cells cultured on different substrata (scale bar, 100 μm). (C) Levels of ppErk and Erk in cells cultured on different substrata, as measured by immunoblotting analysis. Mean ± SD ppErk/Erk levels were normalized to those measured on soft substrata. n = 3 biological replicates. *p < 0.05 and **p < 0.01, paired t test. (D) KTR reports real-time Erk activity in individual cells by localizing to the cytoplasm or nucleus under high or low levels of Erk phosphorylation, respectively. (E) Representative images of KTR-expressing MCF10A cells cultured on different substrata (scale bar, 100 μm). (F) Representative heatmaps of KTR-reported Erk activities for 20 cells on each substratum. Each row of the heatmap represents one cell. (G) Quantification of the time-averaged Erk activity in cells cultured on each substratum. Boxes represent the 25th to 75th percentiles, with mean values indicated by horizontal lines. Whiskers represent the minimum and maximum of each condition. n > 60 cells from three biological replicates. n.s., not significant; ***p < 0.001, one-way ANOVA and Tukey post hoc tests. (H) Distribution of pulses detected in cells cultured on each substratum. Points denote the mean ± SD of three biological replicates. (I) Fractions of cell populations on each substratum exhibiting constantly active (on), pulsing (at least two pulses detected), or constantly inactive (off) Erk dynamics. Error bars denote SD of three biological replicates.

We first set out to characterize Erk dynamics in MCF10A human mammary epithelial cells under a range of normal and patho-physiological mechanical conditions. In their native tissue, mammary epithelial cells may be exposed to microenvironmental stiffnesses ranging from that of soft, normal mammary tissue (elastic modulus *E*_soft_ ~ 0.1 kPa) to stiff mammary tumors (*E*_stiff_ ~ 4 kPa). We therefore cultured cells on soft (*E*_soft_ ~ 0.1 kPa), intermediate (*E*_intermediate_ ~ 0.9 kPa), and stiff (*E*_stiff_ ~ 4 kPa) substrata (Figures 1A and 1B). Changes to substratum stiffness had a striking effect on tissue morphology: cells were rounded and grew in multi-layered clusters on soft substrata but as monolayers in intermediate and stiff microenvironments (Figure 1B). Immunoblotting analysis revealed that the levels of doubly phosphorylated Erk (ppErk) were higher in cells cultured on stiff substrata or tissue culture-grade polystyrene (TCPS) than in cells on soft substrata (Figure 1C), consistent with previous studies (Trappmann et al., 2012; Yang et al., 2018). However, these static population-wide averages mask changes in the dynamics of Erk signaling that occur in single cells.

To determine how the mechanical properties of the microenvironment affect Erk signaling dynamics in individual cells, we stably expressed an Erk kinase translocation reporter (KTR) (Regot et al., 2014); this biosensor is excluded from the nucleus when Erk activity is high (Figure 1D). To control for changes in cell morphology, we quantified Erk activity by measuring the ratio of KTR fluorescence intensity in the cytoplasm to that in the nucleus (C/N ratio) in single cells over time. We monitored KTR-reported Erk dynamics in cells cultured continuously in growth medium on soft, intermediate, and stiff substrata as well as on fibronectin-coated glass (*E*_glass_ ~ 10^9^ Pa) (Figure 1F; Video S1). These time-lapse experiments revealed striking stiffness-dependent differences in signaling, with Erk activity that was higher and more pulsatile with increasing stiffness. While cells on stiffer substrata exhibited a range of Erk signaling amplitudes, cells on soft substrata exhibited consistently low Erk signaling, as revealed by nuclear localization of KTR (Figure 1E; Video S1). Quantifying the time-averaged C/N ratios of individual cells (Figure 1G), as well as population-averaged C/N ratios over time (Figure S1A), confirmed these stark differences in Erk signaling amplitude. Pulses of Erk signaling were only rarely observed in cells on soft substrata, with higher proportions of pulsing and constant-on cells as stiffness was increased (Figures 1H and 1I; Video S1). Erk activity on stiff substrata could be inhibited by treatment with pharmacological inhibitors of EGFR or Mek, suggesting that under these conditions signaling is mediated primarily by EGFR and the canonical Erk pathway (Figures S1B and S1C). Thus, despite identical biochemical culture conditions, substratum stiffness tunes steady-state Erk signaling dynamics over a broad range in mammary epithelial cells.

**Figure 2.**
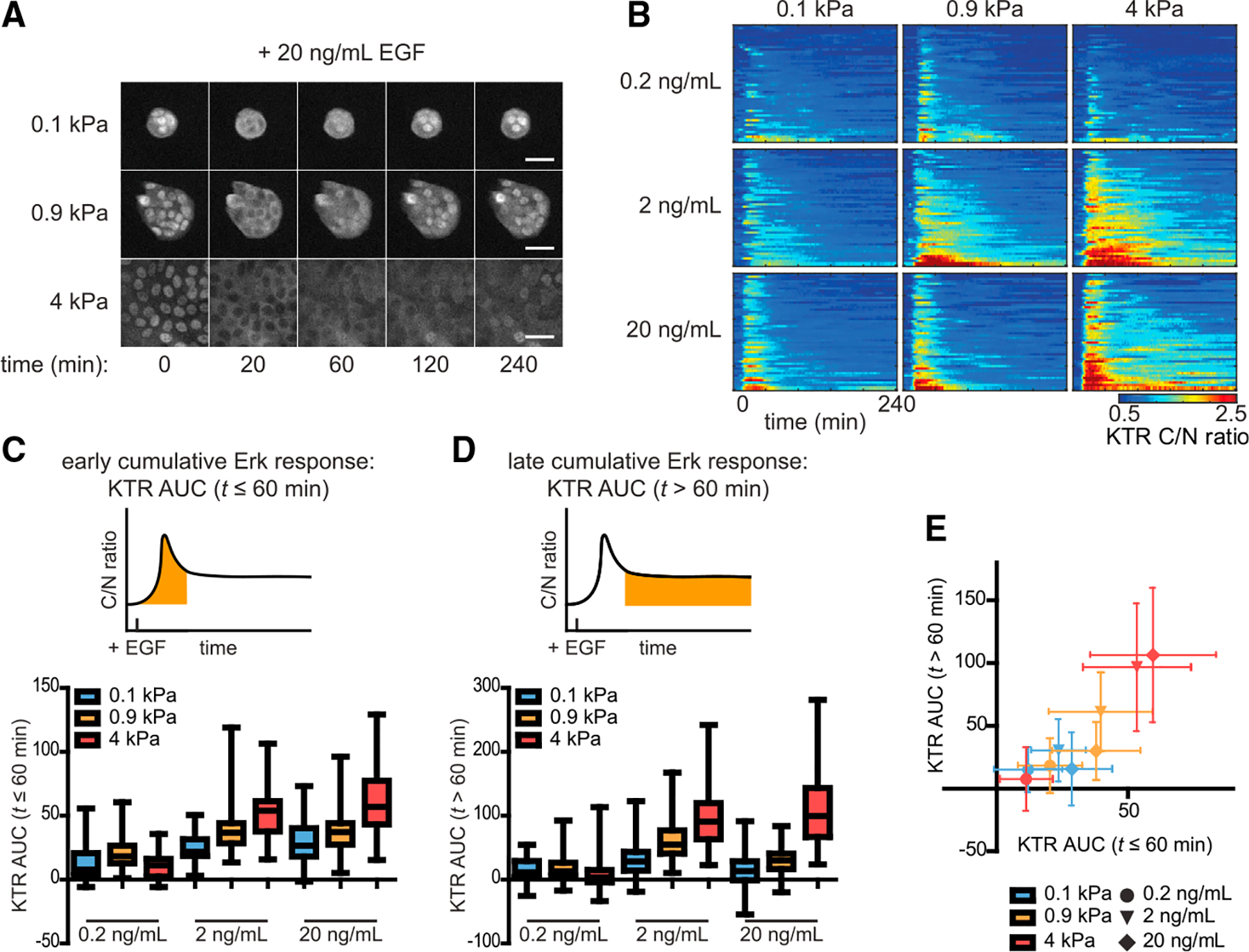
Growth factor-stimulated Erk signal transmission is attenuated by culture on soft substrata (A) Representative time-lapse frames of cells treated with EGF (20 ng/mL) on different substrata (scale bars, 20 μm). (B) Heatmaps of KTR-reported Erk activities for cells on different substrata treated with 0.2, 2, or 20 ng/mL EGF. Each row of the heatmap represents one cell. (C and D) Area under the curve (AUC) of KTR-reported Erk activity in the (C) ≤60 min and (D) >60 min periods after EGF treatment. Boxes and whiskers represent the 25th to 75th percentiles and minima and maxima, respectively. Mean values are indicated by horizontal lines. (E) Mean ± SD late versus early KTR AUCs for each condition. In (B)–(E), n = 50 cells from two biological replicates for each condition.

Classic studies demonstrated that different ligands and growth factor receptors can elicit population-averaged Erk responses ranging from a transient pulse that returns to baseline within 30 min to a sustained response that remains elevated for hours (Bishop et al., 1994; Freed et al., 2017; Marshall, 1995; Santos et al., 2007; Traverse et al., 1994). We thus set out to test whether substratum stiffness also alters the duration of ligand-stimulated Erk signaling. We plated KTR-expressing mammary epithelial cells on substrata of increasing stiffness, cultured them in growth factor (GF)-free media, stimulated with a range of EGF concentrations, and monitored Erk activity using time-lapse microscopy (Figures 2A and 2B; Video S2). In cells in each microenvironment, we observed low levels of Erk activity in the absence of growth factors and a peak of Erk activity with similar kinetics appearing ~15 min after EGF treatment (Figure 2B). Regardless of substratum stiffness, a 0.2 ng/mL dose of EGF induced weak Erk responses that adapted rapidly back to basal levels of Erk signaling, as quantified by the area under the curve (AUC) of KTR-reported Erk activity (Figures 2C and 2D). For 2 and 20 ng/mL doses of EGF, however, increasing substratum stiffness resulted in stronger and more sustained Erk responses (Figures 2C and 2D). Plotting the early and late responses for each dose and substratum revealed that substratum stiffness affects signaling strength on both short and long timescales and that the range of ligand-induced responses increases gradually as substratum stiffness increases (Figure 2E).

MCF10A cells form round, multi-layered clusters when cultured on soft substrata but grow as monolayers on intermediate and stiff substrata, raising the possibility that substratum stiffness might only affect Erk signaling through changes in tissue morphology. However, two comparisons argue against this possibility: the steady-state Erk dynamics on stiff substrata versus glass (Figure 1F) and the EGF responses on intermediate versus stiff substrata (Figure 2B). Cells form two-dimensional (2D) monolayers in each of these conditions yet exhibit different strengths of Erk signaling. As a further test, we measured EGF-stimulated Erk dynamics in isolated, neighbor-less cells versus multicellular clusters on soft substrata (Figure S2A). Treatment with 20 ng/mL EGF produced similarly weak Erk responses in both isolated cells and tissues in soft microenvironments (Figures S2B and S2C), suggesting that the multi-layered tissue morphology caused by soft substrata is not necessary to decrease Erk signaling.

We also tested whether our results generalize to an additional cellular context: primary mouse keratinocytes, an epidermal cell type in which Erk signaling dynamics downstream of growth factor receptors have been extensively characterized (Goglia et al., 2020; Hiratsuka et al., 2015, 2020). Like MCF10A cells, keratinocytes exhibited higher, more sustained levels of Erk activity on stiff substrata compared with soft substrata (Figures S2D–S2G), and a brief transient response was observed even from isolated cells on soft substrata (Figure S2E).

Our data reveal that both steady-state and growth factor-stimulated Erk signaling dynamics depend strongly on the mechanical properties of the microenvironment. When cultured in growth media, cells shifted from low Erk activity on soft substrata to pulsatile or constant activity on stiff substrata. When stimulated with EGF, cells on soft substrata exhibited transient Erk activation, whereas cells on stiffer substrata exhibited a larger range of Erk responses across EGF doses. Thus, for identical biochemical stimuli, the mechanical microenvironment represents a major control point over Erk signaling dynamics, both in steady-state culture and after acute growth factor stimulation.

### Optogenetic Ras and EGFR stimuli drive sustained Erk responses regardless of substratum stiffness

Many molecular links have been identified between the mechanical microenvironment and Erk signaling (Aplin et al., 2001; DeMali et al., 1999; Giancotti and Ruoslahti, 1999; Lin et al., 1996; Renshaw et al., 1997). Which might explain the stiffness-dependent changes we observe in Erk dynamics? We first sought to identify points along the Erk pathway that limit signaling in soft microenvironments by measuring Erk activity in response to optogenetic stimulation at various nodes. Specifically, we reasoned that if stiffness-dependent differences in Erk are still observed when stimulating the pathway at an intermediate node, crosstalk from the mechanical microenvironment to the Erk pathway is likely to act between the intermediate node and Erk.

**Figure 3.**
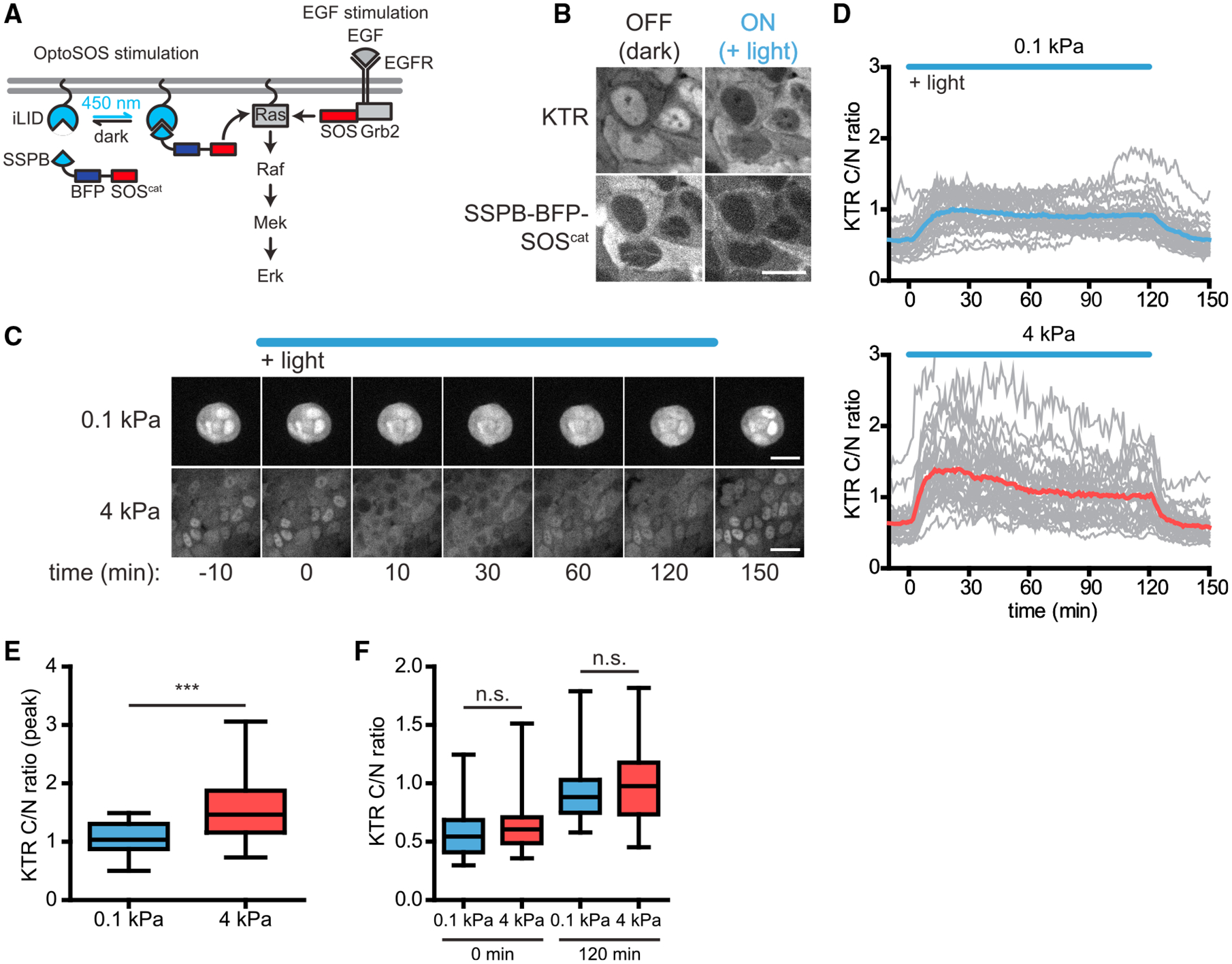
Ras-level stimulation of Erk signaling produces sustained Erk dynamics regardless of substratum stiffness (A) The OptoSOS system recruits the SOS catalytic domain through the blue light-responsive iLID/SSPB heterodimerizing protein pair to stimulate membrane-bound Ras. (B) Representative images of OptoSOS-expressing MCF10A cells before and after OptoSOS stimulation (scale bar, 15 μm). (C) Representative time-lapse frames of cells stimulated with OptoSOS on soft or stiff substrata (scale bars, 30 μm). (D) Mean Erk trajectories in response to 120 min continuous OptoSOS stimulation, with the responses of individual cells represented by lighter gray lines. (E) Peak response of Erk activity following OptoSOS stimulation. (F) C/N ratios before and 120 min after OptoSOS stimulation. Boxes and whiskers in (E) and (F) represent the 25th to 75th percentiles and minima and maxima, respectively. Mean values are indicated by horizontal lines. In (D)–(F), n = 30 cells from three biological replicates for each condition. ***p < 0.001, unpaired t test; n.s., not significant.

We first generated MCF10A cells expressing both the KTR and the OptoSOS system (Goglia et al., 2020), in which blue light is used to recruit an activator of Ras (SOS^cat^) to the membrane (Figures 3A and 3B). We cultured cells on soft or stiff substrata in the absence of growth factors, continuously stimulated with blue light for 120 min, and tracked the resulting Erk dynamics (Figures 3C and 3D; Video S3). In both microenvironments, blue light illumination drove a rapid increase in Erk activity that was sustained if light was present and returned to a baseline state minutes after light removal (Figures 3C and 3D). Long-term light stimulation caused Erk activity to reach a steady-state level that did not vary with substratum stiffness and did not adapt back to the pre-stimulus baseline as observed after EGF stimulation (Figures 3D and 3F). A second feature of the light-induced response, the initial peak amplitude, was higher in cells on stiff substrata, suggesting that the mechanical microenvironment exerts some degree of control over initial pulse amplitude between Ras and Erk, although it remains possible that the apparent KTR peak height is influenced by cell morphology differences between these conditions (Figure 3E). Nevertheless, these data demonstrate that Ras-level stimulation is sufficient to generate a sustained Erk signal even in soft microenvironments, suggesting that a major regulatory link between substratum stiffness and Erk activity lies upstream of Ras.

**Figure 4.**
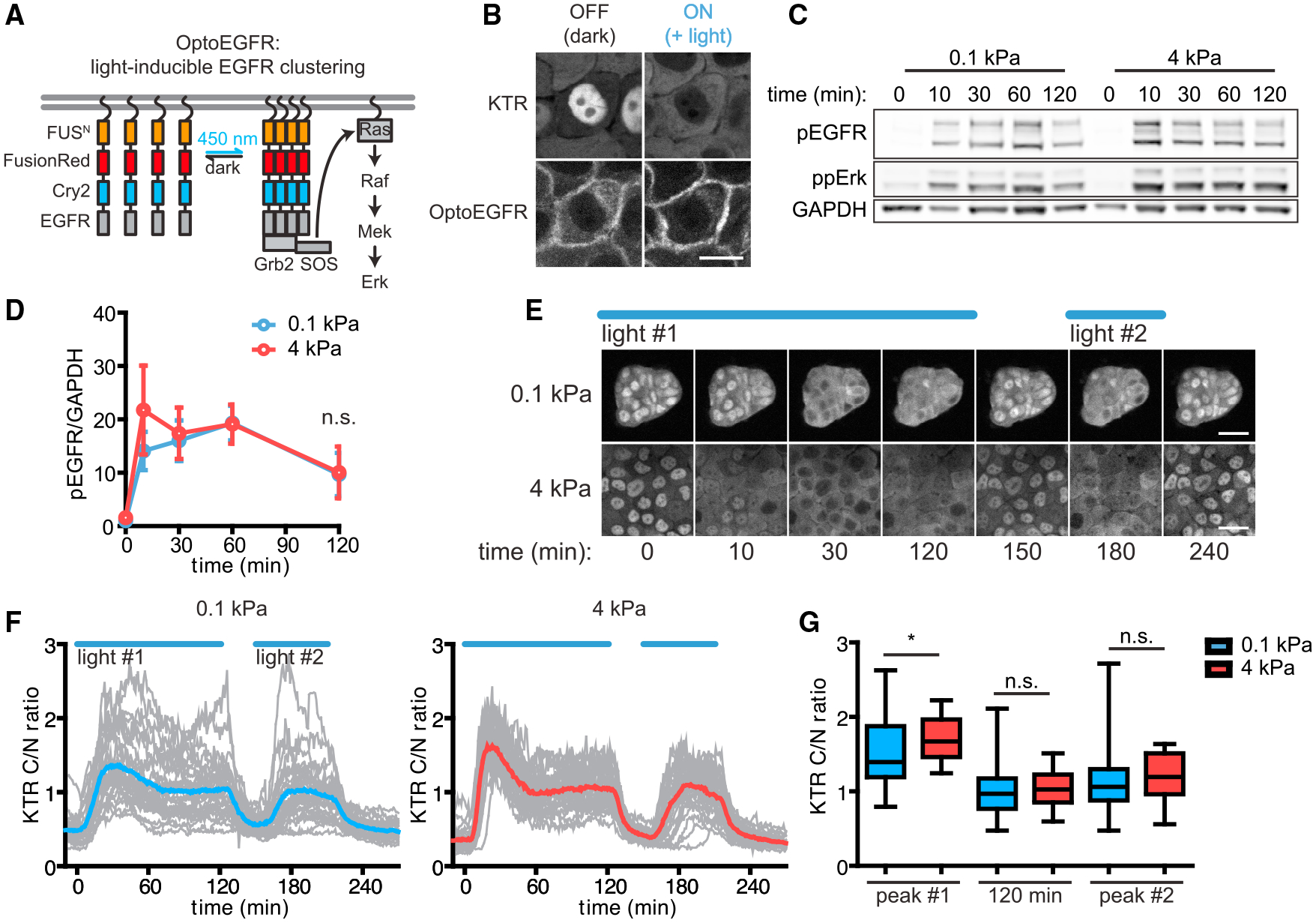
Signal transmission downstream of receptor clustering is unaffected by the stiffness of the microenvironment (A) Blue light-induced clustering of membrane-bound EGFR cytosolic domains leads to their autophosphorylation and signal transmission through the EGFR/Erk pathway. (B) Representative images of OptoEGFR-expressing cells before and after OptoEGFR stimulation (scale bar, 15 μm). (C) OptoEGFR phosphorylation levels before and after OptoEGFR stimulation in cells on soft or stiff substrata, as measured by immunoblotting analysis. Bottom and top bands of pEGFR immunoblots are OptoEGFR, and the middle bands are endogenous EGFR. (D) Quantification of the immunoblots from (C). Points denote mean ± SEM of three biological replicates. n.s., not significant, unpaired t test. (E) Representative time-lapse frames of cells stimulated with OptoEGFR on soft or stiff substrata (scale bars, 30 μm). (F) Mean Erk trajectories in response to sequential 120 and 60 min periods of continuous OptoEGFR stimulation, with the responses of individual cells represented by gray lines. (G) Peak C/N ratios after each OptoEGFR stimulation period and C/N ratios 120 min after the first period of OptoEGFR stimulation. *p < 0.05, unpaired t test; n.s., not significant. Boxes and whiskers represent the 25th to 75th percentiles and minima and maxima, respectively. Mean values are indicated by horizontal lines. In (E)–(G), n = 30 cells from three biological replicates for each condition.

We next tested for stiffness-dependent signaling differences further upstream, between EGFR and Ras. We generated an optogenetic tool (OptoEGFR) on the basis of recent successes using light-induced clustering to activate the cytosolic domains of receptor tyrosine kinases without requiring exogenous ligands (Dine et al., 2018; Kim et al., 2014). We fused the cytosolic domain of EGFR to a myristoylation-based membrane localization tag, the FusionRed fluorescent protein, and the OptoDroplet system (Shin et al., 2017), such that blue light could trigger both the clustering and autophosphorylation of EGFR cytosolic domains (Figures 4A and 4B). Immunoblotting lysates from light-stimulated OptoEGFR-expressing cells on soft and stiff substrata confirmed that light triggered OptoEGFR phosphorylation to similar levels and with similar kinetics regardless of the mechanical properties of the microenvironment (Figures 4C and 4D).

After generating MCF10A cells co-expressing OptoEGFR and the KTR, we monitored light-induced Erk signaling in cells cultured on soft and stiff substrata. Our results closely mirrored the results obtained by OptoSOS stimulation: we observed a higher initial peak of Erk activity in cells on stiff substrata relative to those on soft substrata, followed by a comparable plateau of long-term Erk activity regardless of substratum stiffness (Figures 4E–G, Video S4). We also observed that cells rapidly returned to low baseline Erk activity upon removal of the light stimulus, and a second light pulse drove cells back to the steady-state signaling level without a second stiffness-dependent transient peak (Figures 4F and 4G). This result suggests that stiffness-dependent differences in peak Erk activation are relatively limited, only affecting the first stimulus after prolonged starvation. Taken together, these data demonstrate that prolonged activation of either EGFR cytosolic domains or the Ras GTPase trigger sustained Erk activity at a similar level regardless of substratum stiffness. It is thus likely that the major stiffness-associated differences in Erk signaling dynamics act upstream of the cytosolic receptor domains, potentially by altering EGFR expression levels and/or the efficiency with which the receptor is activated by extracellular ligands.

### Substratum stiffness modulates EGFR expression, activation, and ligand-receptor binding

**Figure 5.**
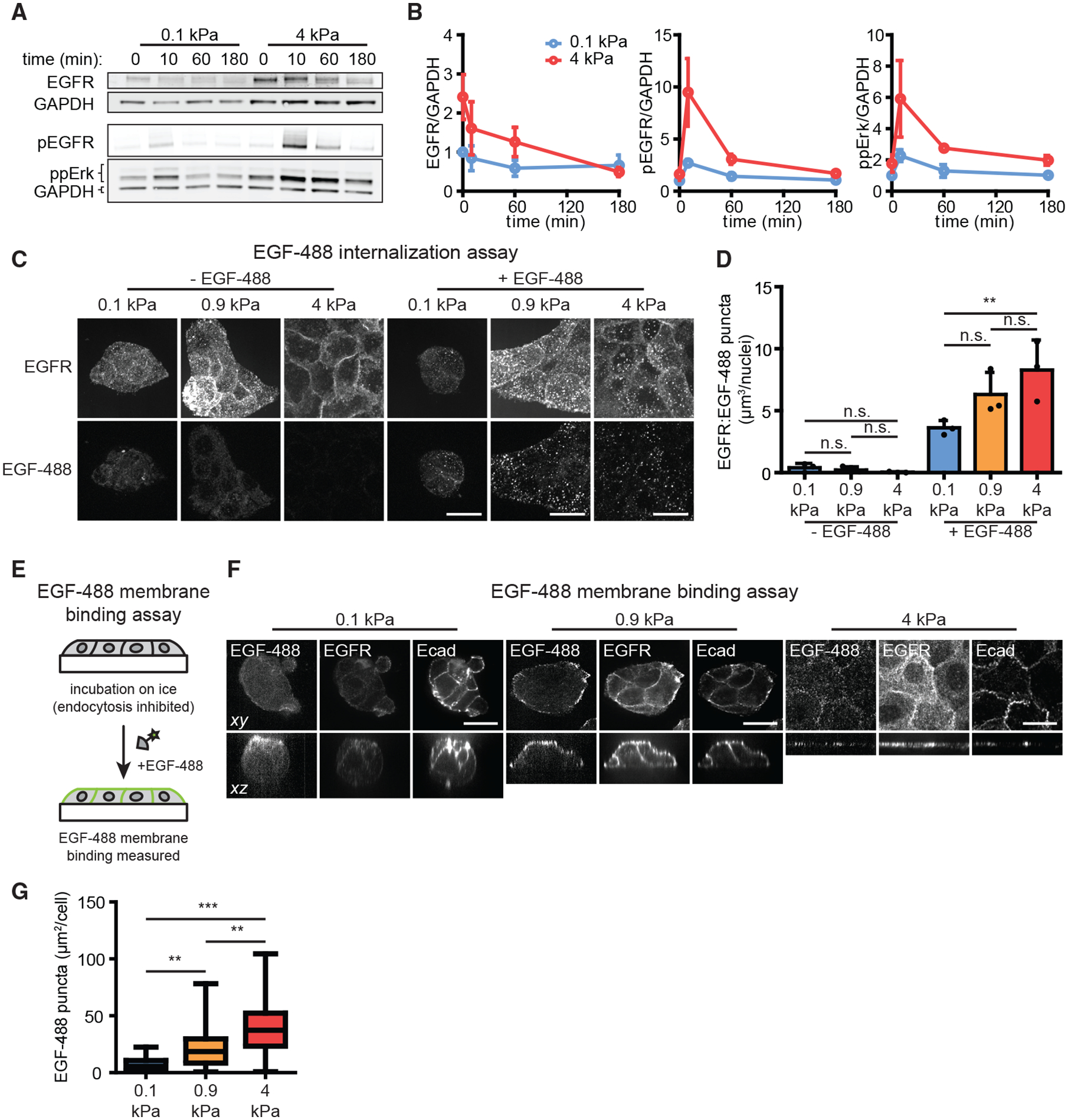
Soft substrata decrease EGFR activation and ligand-receptor binding (A) MCF10A cells on soft or stiff substrata were treated with EGF (20 ng/mL) for different amounts of time and subjected to immunoblotting for phosphorylated EGFR (pEGFR), ppErk, and total EGFR. (B) Quantification of the immunoblots from (A). Points denote mean ± SEM of three biological replicates. (C) Representative maximum-intensity projection (max IP) images of cells treated with EGF-488 (20 ng/mL) for 10 min and subjected to immunostaining analysis for EGFR (scale bars, 20 μm). (D) Mean + SD volume of puncta doubly positive for EGFR and EGF-488 from (C). Points denote mean values from three biological replicates. n.s., not significant; **p < 0.01, one-way ANOVA and Tukey post hoc tests. (E) To measure EGF binding at the cell surface, samples were cultured on ice to inhibit endocytosis, treated with EGF-488 (20 ng/mL), and subjected to fixation for imaging analysis. (F) Representative images of cells on different substrata subjected to EGF-488 membrane binding assays and immunostaining for EGFR and E-cadherin (Ecad) (scale bars, 20 μm). (G) Quantification of the area of EGF-488 puncta per cell from (F). Boxes and whiskers represent the 25th to 75th percentiles and minima and maxima, respectively. Mean values are indicated by horizontal lines. For each condition, n > 25 cells from three biological replicates. **p < 0.01 and ***p < 0.001 using one-way ANOVA and Tukey post hoc tests.

Our optogenetic perturbations suggest that substratum stiffness may affect Erk signal transmission at the top-most steps of the pathway, altering the expression level of EGFR and/or its ability to be activated by extracellular ligands. To test this hypothesis, we first measured the levels of total EGFR, Tyr1068-phosphorylated EGFR (pEGFR), and ppErk over time in MCF10A cells that were cultured on soft or stiff substrata, swapped to GF-free media, and treated with EGF (Figure 5A). Prior to EGF treatment, the total levels of EGFR were modestly (~2-fold) higher in cells cultured on stiff substrata; EGF treatment then triggered a decrease in total EGFR on stiff substrata until levels were comparable in both microenvironments (Figure 5B, left panel). In contrast, the level of pEGFR exhibited a higher (~3.5-fold) peak in cells cultured on stiff substrata than soft substrata (Figure 5B, middle panel), indicating that a greater proportion of EGFR was phosphorylated in cells in stiff microenvironments. ppErk levels remained higher on stiff substrata throughout the time course (Figure 5B, right panel), consistent with our live-cell KTR imaging and likely reflecting additional amplification within the pathway between EGFR and Erk (Goglia et al., 2020; Pinilla-Macua et al., 2017). Overall, these data reveal stiffness-dependent differences in both total EGFR protein levels and the efficiency of EGFR activation, consistent with multiple forms of receptor-level control by the mechanical microenvironment.

As a second test of stiffness-dependent differences in EGFR activation, we set out to directly measure how EGF binding and internalization varied with substratum stiffness. We treated cells on soft, intermediate, and stiff substrata with a fluorescently labeled EGF (EGF-488), fixed cells, and subjected them to immunofluorescence analysis to simultaneously assess receptor and ligand localization. Prior to treatment with EGF-488, EGFR was primarily localized at cell membranes in each case (Figure 5C). Treatment with 20 ng/mL EGF-488 for 10 min led to higher levels of receptor and ligand internalization for cells on stiffer substrata, as quantified by the volume of puncta per cell containing both EGFR and EGF-488 (Figure 5D). Greater amounts of internalized EGFR persisted on stiff substrata hours after stimulation even while total EGFR protein levels decreased (Figure S3A), matching the timescale of sustained Erk activity observed above (Figure 2). Treatment with kinase inhibitors against EGFR or Mek further confirmed that EGFR and EGF-488 internalization required EGFR kinase activity but not Erk activity (Figure S3B), consistent with prior reports that receptor internalization depends on receptor activity (Wiley et al., 1991). Taken together, these results confirm that stiff microenvironments enhance EGFR signaling beyond simply increasing EGFR expression levels.

How might the efficiency of ligand-stimulated EGFR signaling be attenuated in soft microenvironments? We reasoned that soft microenvironments might (1) directly interfere with ligand-receptor binding at the cell surface or (2) leave ligand-receptor binding unaffected but interfere with subsequent EGFR activation. To discriminate between these possibilities, we set out to measure EGF-488 binding 10 min after stimulation under conditions where endocytosis is inhibited by incubating cells on ice (Figure 5E) (Liang et al., 2008). Performing this assay in the presence or absence of kinase inhibitors revealed small clusters of cell-surface-bound EGF-488 whose spatial distribution was unaffected by signaling through EGFR or Mek (Figure S3C). We then performed the assay on cells cultured on different substrata, which revealed markedly different patterns of EGF-488 binding in each case (Figures 5F and S3D). EGF-488 puncta were evenly distributed across the cell surface on stiff substrata, matching our observations of cells cultured on glass (Figure 5F, right panels). In contrast, EGF-488 binding was largely absent from cell-cell contacts for cells cultured on intermediate substrata, despite exhibiting uniform E-cadherin and EGFR levels (Figure 5F, middle panels). Finally, EGF-488 binding was dramatically reduced on both the cell-cell and cell-media interfaces of cells cultured on soft substrata (Figure 5F, left panels). Quantifying the area of EGF-488 puncta observed per cell revealed an overall ~6-fold difference in EGF-488 puncta between soft and stiff substrata (Figure 5G). These data suggest that the same concentration of soluble EGF results in substantially different binding depending on substratum stiffness, pinpointing the top-most step in growth factor signaling—ligand-receptor interactions—as a major node affected by substratum stiffness.

### Substratum stiffness modulates Erk signaling independently of the mechanosensitive transcription factor YAP

The canonical mechanosensor Yes-associated protein (YAP) has been shown to enhance the transcript levels of EGFR by binding to its enhancer (Zanconato et al., 2015) and proximal promoter (Song et al., 2015) regions. Culture in stiff microenvironments promotes YAP nuclear localization, which subsequently results in the activation of YAP-target genes (Aragona et al., 2013). We therefore hypothesized that the decreased EGFR levels in cells on soft substrata are caused by decreased YAP nuclear localization (Figure S4A) and lower gene expression. To test this hypothesis, we used quantitative real-time PCR (qRT-PCR) analysis to measure the transcript levels of EGFR in MCF10A cells cultured on soft and stiff substrata. Surprisingly, we were unable to detect differences in EGFR transcripts (Figure S4B) despite pronounced differences in protein levels (Figure 5B). To test whether YAP is necessary for EGFR expression, we generated a stable cell line that expressed YAP-targeting short hairpin RNA (shRNA) under the control of a doxycycline-inducible promoter (TetON-shYAP) (Figure S4C) (Er et al., 2018). Consistent with the results of our transcript analysis, we found that decreasing YAP expression had no noticeable effect on the levels of EGFR protein in cells cultured on TCPS (Figure S4C). Furthermore, treating cells with EGF and monitoring KTR revealed similar Erk dynamics in both the shYAP and control cells, with no measurable effect on the duration of signaling (Figures S4D and S4E). Conversely, ectopically expressing a constitutively active YAP (YFP-YAP^5SA^) (Ege et al., 2018) in cells on soft substrata did not significantly alter the levels of EGFR (Figure S4F) or ppErk (Figure S4G). These findings suggest that substratum stiffness regulates both Erk signaling and EGFR protein expression in MCF10A cells through mechanisms independent of YAP-induced transcriptional activation.

### Ectopic EGFR expression drives stiff-like Erk signaling in soft microenvironments

**Figure 6.**
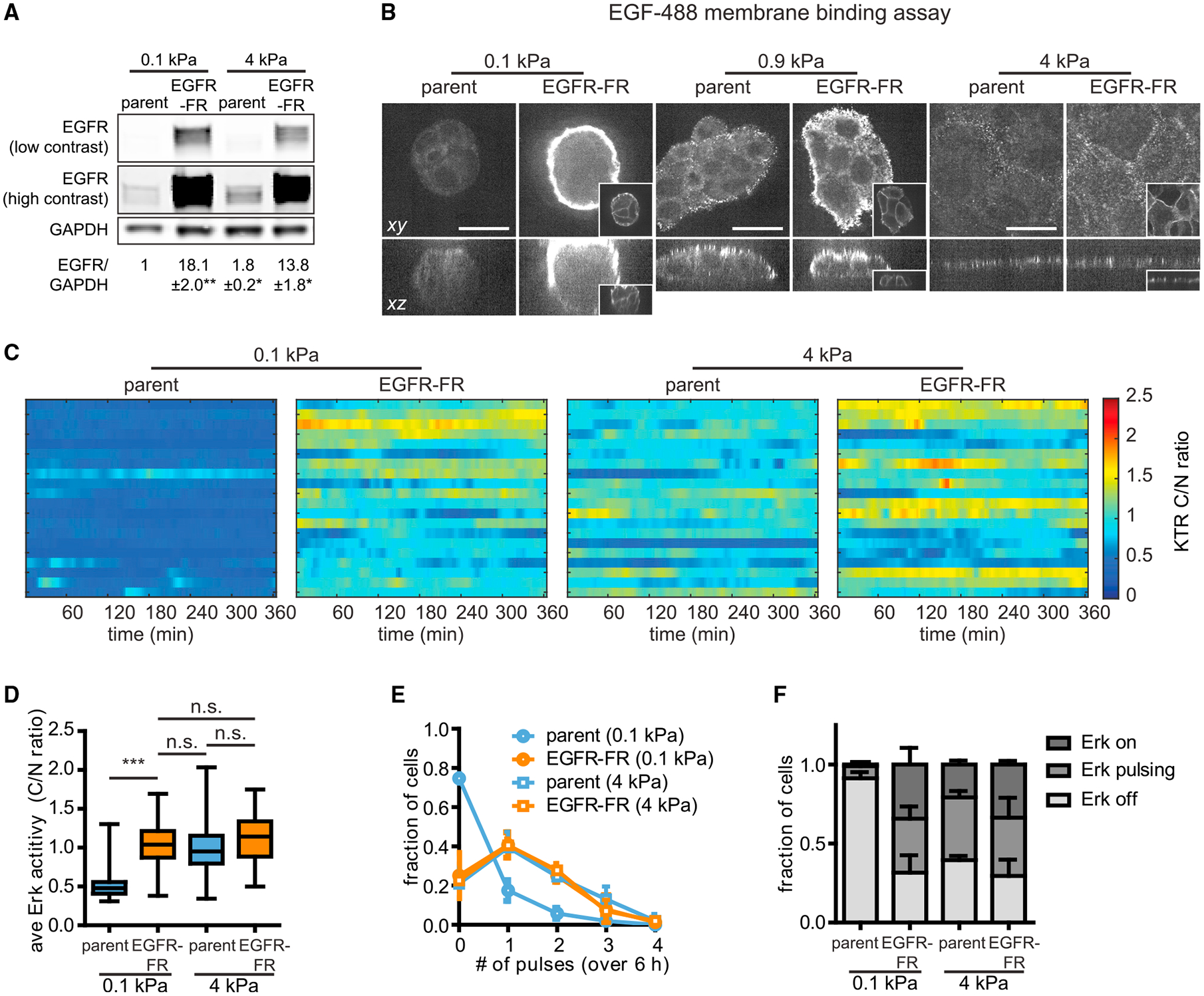
Ectopic expression of EGFR amplifies Erk signaling in cells on soft substrata (A) Immunoblotting analysis and quantification of EGFR protein levels in EGFR-FR cells or the parental MCF10A cell line. n = 3 biological replicates. *p < 0.05 and **p < 0.01, paired t test. (B) Representative EGF-488 images of EGFR-FR cells or parental cells subjected to EGF-488 membrane binding assays. Inset images display EGFR-FR in EGFR-FR cells (scale bars, 20 μm). (C) Representative heatmaps of KTR-reported Erk activities for EGFR-FR or control cells cultured on soft or stiff substrata in the presence of growth medium. Each row of the heatmap represents one cell. (D) Quantification of the time-averaged Erk activity in cells cultured on each substratum. Boxes represent the 25th to 75th percentiles, with mean values indicated by horizontal lines. Whiskers represent the minimum and maximum of each condition. For each condition, n > 100 cells from three biological replicates. n.s., not significant; ***p < 0.001, one-way ANOVA and Tukey post hoc tests. (E) Distribution of pulses detected in cells cultured on each substratum. Points denote the mean ± SD of three biological replicates. (F) Fractions of cell populations on each substratum exhibiting constantly active (on), pulsatile (at least two pulses detected), or constantly inactive (off) Erk dynamics. Error bars denote SD of three biological replicates.

Having observed that soft microenvironments limit EGFR signaling at the level of ligand-receptor binding, we next tested whether increasing EGFR expression could be sufficient to increase Erk signaling in soft microenvironments. EGFR expression levels vary over orders of magnitude between cell types (Herbst, 2004) and are commonly elevated in tumor cells (Sanchez-Vega et al., 2018), so it is important to know whether simply altering EGFR expression is sufficient to override stiffness-related modulation. We thus generated an MCF10A cell line co-expressing KTR and a FusionRed-tagged EGFR (EGFR-FR) (Figure 6A). EGFR-FR MCF10A cells exhibited similar morphology to the parental cell line, forming clusters on soft substrata and monolayers under intermediate and stiff conditions (Figure 6B).

We cultured EGFR-FR cells or the parental cell line on different substrata and compared EGF-488 ligand binding at the membrane between these microenvironments (Figure 5E). Relative to the parental cell line, EGFR-FR cells on soft substrata exhibited a striking increase in EGF-488 binding at the periphery of tissues yet still lacked any observable EGF-488 binding at sites of cell-cell contact (Figures 6B and S5A, left panels). Although EGFR overexpression also increased EGF-488 binding at the periphery of tissues on intermediate substrata (Figures 6B and S5A, middle panels), increasing receptor expression appeared to have surprisingly little effect on EGF-488 binding on stiff substrata, where we observed a similar number of EGF-488 puncta as the parental cell line (Figures 6B and S5A, right panels). Similarly, EGFR overexpression did not significantly alter the amount of EGF-488 internalization in cells on stiff substrata but drastically increased ligand internalization in cells on soft substrata (Figures S5B and S5C).

Given that ectopic EGFR expression is sufficient to increase ligand binding and internalization, we next tested whether the effects extended to downstream Erk signaling. We cultured EGFR-FR cells and control cells on soft substrata in the absence of growth factors, then treated with EGF and monitored Erk activity (Figure S5D). Again, we found that parental cells exhibited a transient pulse of Erk activation on soft substrata. In contrast, EGFR-FR cells exhibited a sustained Erk response, remaining elevated 120 min after EGF stimulation (Figures S5E and S5F). Finally, we examined steady-state Erk signaling dynamics in EGFR-FR or parental cells cultured in growth medium on soft or stiff substrata (Figure 6C; Video S5). Quantification of single-cell signaling revealed that EGFR-FR cells exhibited elevated amplitudes and frequencies of pulsatile Erk dynamics on soft substrata, shifting their dynamics to be indistinguishable from those of parental or EGFR-FR cells on stiff substrata (Figures 6D–6F). From these data we conclude that increasing EGFR protein levels can indeed overcome the attenuated Erk signaling downstream of growth factor receptors in soft microenvironments. Interestingly, these effects appear to be buffered on stiffer substrata, where EGFR overexpression had minimal effects on both ligand binding and downstream signaling.

## DISCUSSION

Here, we reveal how the mechanical microenvironment alters Erk signaling dynamics and identify mechanically regulated processes in the EGFR/Erk pathway. We find that increasing substratum stiffness elicits a dramatic shift in Erk dynamics, from a quiescent, inactive state to pulses of activity in growth media (Figure 1), and from a transient response to sustained activation after EGF stimulation (Figure 2). By combining mechanically tunable substrata with optogenetic tools targeting multiple nodes, we find that the intracellular pathway between EGFR and Erk is relatively insensitive to changes in substratum stiffness (Figures 3 and 4). In contrast, we find that both the expression of EGFR and efficiency of its activation differ substantially between soft and stiff substrata (Figure 5), pinpointing the source of mechanically regulated Erk signaling in mammary epithelial cells.

Growth factor signaling and the mechanical properties of the microenvironment cooperatively regulate tissue function (Sarker et al., 2020). In normal tissues, soft microenvironments might serve to dampen mitogenic signaling to maintain tissue homeostasis. Conversely, abnormally stiff microenvironments might heighten the sensitivities of cells to mitogens, leading to uncontrolled proliferation and invasiveness (Levental et al., 2009; Paszek et al., 2005). We show that the mechanical microenvironment can tune the range of information transmitted through a biochemical pathway, funneling different EGF doses to a similar response on soft substrata and eliciting distinct responses on stiffer substrata (Figure 2). Thus, as signaling dynamics begin to be interrogated within complex systems such as organoids (Muta et al., 2018), embryos (Pokrass et al., 2020; Simon et al., 2020), and mature organisms (Hiratsuka et al., 2020), both biochemical and mechanical aspects of the microenvironment should be taken into consideration.

Our study also offers an experimental workflow to begin teasing apart how mechanical and biochemical cues are jointly interpreted. By delivering controlled inputs to different pathway nodes and measuring responses, we characterize signal transmission through entire sections of the pathway at once. If similar responses are observed between mechanical microenvironments, then no mechanically regulated change within that section of the pathway is necessary to explain the overall difference. We are thus able to report that the full cytosolic pathway, from active EGFR to Erk, can drive constant, high levels of Erk activation as long as an intracellular stimulus is present (Figures 3 and 4). We do find differences in peak Erk activity ~30 min after stimulation, indicating that stiffness-dependent differences are present within the pathway but that their effects are felt transiently during the first stimulus after prolonged starvation. Nevertheless, these differences are not sufficient to explain the hours-scale differences in signaling strength observed upon EGF stimulation of cells on soft and stiff substrata. One important implication of these results is that soft substrata do not create “bottlenecks” that limit signal transmission within the intracellular EGFR/Erk pathway. Our results would predict that activating mutations at any intracellular EGFR/Erk pathway node would be capable of driving pathologically high Erk activity, regardless of mechanical context.

If the intracellular pathway performs similarly across substrata of varying stiffness, then how does substratum stiffness tune growth factor signaling? Our data point to multiple mechanisms that regulate the top-most step in the pathway: receptor activation by the EGF ligand. First, we observe modestly higher levels of EGFR expression in unstimulated cells on stiff compared with soft substrata (Figures 5A, 5B, and 6A). Similar changes in EGFR expression have also been observed in glioblastoma cells (Umesh et al., 2014) as well as in squamous cell carcinoma (Grasset et al., 2018) to increase growth factor-induced tumor invasion. Second, imaging binding and internalization of fluorescently labeled EGF-488 revealed even larger stiffness-dependent differences, with stiff substrata exhibiting more initial EGF-488 binding and a greater, more persistent pool of internalized EGFR compared with soft substrata (Figure 5 and S3). Finally, we observed stark exclusion of EGF-488 from cell-cell contacts on intermediate and soft substrata despite uniform EGF localization around the cell periphery (Figure 6B), suggesting that changes in tissue morphology caused by the mechanical microenvironment play a significant role in receptor-level signaling. On the basis of these observations, we propose that substratum stiffness primarily regulates Erk signaling dynamics at the top-most step in the pathway, modulating the extent, location, and efficiency of ligand-receptor activation.

Of these three receptor-level mechanisms—shifts in EGFR expression, exclusion of EGF from cell-cell contacts, and changes in EGF binding—the change in EGF binding was the most surprising and, we argue, is likely to be the most important when cells experience changes in the mechanical microenvironment. Stiffness-associated differences in EGFR expression alone cannot fully explain our results, as we observe a larger fold change in EGF binding (~6-fold; Figure 5G) and pEGFR (~3.5-fold; Figure 5B) between soft and stiff substrata than we do in total EGFR levels (~2-fold; Figure 5B). Also, differences in Erk signaling persist even at time points wherein differences in EGFR protein levels are no longer observed (Figures 2, 5A, and 5B). Although overexpressing EGFR can shift cells on soft substrata to a stiff-like response, these results reflect a ~15-fold increase in EGFR levels, far exceeding the ~2-fold difference between isogenic cells in soft and stiff conditions. EGFR overexpression also does not drive a corresponding increase in EGF-488 binding on stiff substrata (Figure 6B), further suggesting that ligand-receptor interactions cannot be explained solely by receptor levels. Likewise, EGF exclusion from cell-cell contacts cannot explain the attenuated Erk signaling of isolated cells on soft substrata (Figures S2A–S2C). In contrast, we observe stark differences in EGF-488 binding between cells on soft and stiff substrata (Figures 5F and 5G) that correlate well with Erk activity in all scenarios (Figures 5 and 6).

Our results and interpretation point to the next mechanistic challenge: explaining how the mechanical microenvironment alters ligand-receptor interactions. Many plausible mechanisms have been proposed, including sequestration of EGFR to inactive sub-compartments of the plasma membrane (Chiasson-MacKenzie and McClatchey, 2018) and modification of the receptor (e.g., glycosylation) to alter these properties (Kaszuba et al., 2015). It also remains unknown whether our results are specific to EGF and EGFR or whether they might extend to other receptor tyrosine kinases or additional families of surface receptors. For example, EGFR is known to heterodimerize with other ErbB family members, and cellular systems in which these interactions are systematically varied as a function of substratum stiffness could lead to additional insights. We anticipate that our findings may motivate future studies aimed at deciphering how the mechanical properties of the microenvironment alter interactions between EGFR, and potentially other receptors, with their cognate ligands to orchestrate cellular processes throughout tissue development, homeostasis, and disease progression.

### Limitations of the study

Substratum stiffness alters a multitude of cellular properties aside from EGFR/Erk signaling, including overall cell morphology and tissue organization. Mammary epithelial cells formed rounded, multi-layered tissues on soft substrata while spreading to form monolayers on stiffer substrata. As a result, it remains very difficult to decouple effects caused by direct cellular sensing of the mechanical microenvironment from those triggered by changes in cell/tissue morphology. Our study is no exception: measurements of EGF binding to the cell surface (Figures 5F and 6B) suggest that the rounded, multi-layered morphology of tissues in soft microenvironments attenuates signaling by decreasing ligand-receptor binding. On the other hand, we still observe decreased Erk signaling in cells on soft substrata when isolated from neighbors (Figures S2A–S2C). Taken together, our results suggest that the mechanical properties of the microenvironment regulate Erk signaling through both morphology-dependent and morphology-independent mechanisms. Future studies would thus benefit greatly from cell culture platforms that can alter substratum stiffness independently of cell and tissue morphology.

## STAR★METHODS

### RESOURCE AVAILABILITY

#### Lead contact

Further information and requests for resources and reagents should be directed to and will be fulfilled by the lead contact, Jared Toettcher (toettcher@princeton.edu).

#### Materials availability

- Plasmids generated in this study have been deposited to Addgene (www.addgene.org/Jared_Toettcher). Catalog numbers are listed in the key resources table.
- All cell lines produced in this study will be made available upon request.

#### Data and code availability

- All data reported in this paper will be shared by the lead contact upon request.
- MATLAB scripts for the analyses of KTR time-lapses, EGF internalization assays, and EGF membrane binding assays have been deposited at the Toettcher Lab GitHub page and are publicly available as of the date of publication. DOIs are listed in the Key Resources table.
- Any additional information required to reanalyze the data reported in this paper is available from the lead contact upon request.

### EXPERIMENTAL MODEL AND SUBJECT DETAILS

#### Cell culture

MCF10A-5E cells (Janes et al., 2010) were cultured in DMEM/F12 medium (Gibco) supplemented with 5% horse serum (ATCC), 20 ng/mL EGF (R&D Systems), 0.5 μg/mL hydrocortisone (Corning), 100 ng/mL cholera toxin (Sigma-Aldrich), 10 μg/mL insulin (Sigma-Aldrich), and 50 μg/mL penicillin/streptomycin/glutamine (Gibco) (growth medium). Under growth factor-free conditions, cells were cultured in DMEM/F12 medium supplemented with 0.5 μg/mL hydrocortisone, 100 ng/mL cholera toxin, 3 mg/mL bovine serum albumin, and 50 μg/mL penicillin/streptomycin/glutamine (GF-free medium). Dorsal epidermal keratinocytes derived from CD1 mice and expressing a retrovirally-delivered H2B-RFP (obtained from the Devenport Lab) were lentivirally transduced with iRFP-KTR and cultured in DMEM/F12 (3:1) without Ca^2+^ (Life Technologies) supplemented with 15% FBS, 31 mM sodium bicarbonate (Sigma Aldrich), 5 μg/mL insulin (Sigma Aldrich), 0.45 μg/mL hydrocortisone (Sigma Aldrich), 5 μg/mL transferrin (Sigma Aldrich), 0.1 nM cholera toxin (Sigma Aldrich), and 0.2 nM T3 (Sigma Aldrich) (low calcium E medium). Cells were maintained at 37°C and 5% CO_2_.

For experiments on polyacrylamide substrata, cells were seeded at ~40,000 cells/cm^2^ (0.1 kPa and 0.9 kPa substrata) or ~20,000 cells/cm^2^ (4 kPa substrata). MCF10A cells were cultured in growth medium for 24 h before replacing with GF-free or fresh growth medium. Keratinocytes were cultured in low calcium E medium for 24 h before replacing with high calcium E medium (low calcium E medium supplemented with 1.5 mM CaCl_2_). Cells were then analyzed 24 h later.

### METHOD DETAILS

#### Synthetic substrata

To prepare polyacrylamide substrata, 1.5 mm-thick glass coverslips were pre-treated with glutaraldehyde. First, coverslips were treated with 0.1 N NaOH for 30 min, followed by rinsing with deionized water and air drying. Coverslips were then treated with 2% aminopropyltrimethoxysilane (Sigma Aldrich) in acetone for 30 min, washed three times with acetone, and left to air dry. Lastly, coverslips were treated with 0.5% glutaraldehyde (Sigma Aldrich) in PBS for 30 min, washed with deionized water, and left to air dry. For time-lapse imaging experiments, custom glass-bottom dishes were prepared by replacing the bottoms of 35 mm TCPS dishes with glutaraldehyde-treated coverslips, which were sealed using PDMS (Sigma Aldrich).

Acrylamide solution in deionized water (see synthetic substrata composition) was pipetted onto a glutaraldehyde-treated coverslip, sandwiched with an untreated coverslip, and allowed to gel for 1 h at room temperature. The untreated coverslip was then removed, leaving a polyacrylamide hydrogel attached to the glutaraldehyde-treated coverslip. To coat polyacrylamide substrata with fibronectin, substrata were first washed with ethanol, washed three times with PBS, then washed once with HEPES buffer (50 mM, pH 8.5). 1 mg/mL sulfo-SANPAH (Thermo Fisher Scientific) in deionized water was pipetted onto the hydrogel, which was then subjected to UV crosslinking (2.8 J of 365 nm light exposure over 10 min). Substrata were then rinsed once with HEPES and treated again with sulfo-SANPAH and UV crosslinking. Substrata were rinsed three times with HEPES, coated with 100 μg/mL fibronectin (Corning) in PBS, and left at 4°C overnight before seeding cells the next day.

To prepare fibronectin-coated glass, wells of black-walled 96-well plates (Cellvis) were incubated with 10 μg/mL fibronectin dissolved in phosphate-buffered saline (PBS) at 37°C for 40 min. Fibronectin-coated wells were rinsed once with PBS before seeding cells.

#### Synthetic substrata composition

**Table:** 

Acrylamide (% v/v)	Bis-acrylamide (% v/v)	TEMED (% v/v)	APS (% v/v)	Elastic modulus (kPa)
5	0.01	0.05	0.05	0.1
5	0.03	0.05	0.05	0.9
5	0.35	0.05	0.05	4

#### Plasmids and lentiviral production

All plasmids were constructed using InFusion cloning (ClonTech Laboratories) to ligate PCR products to a pHR vector that was opened using PCR. Lentiviruses were produced as reported previously (Goglia et al., 2017). Briefly, lenti-X HEK 293T cells were co-transfected with pCMV-dR8.91, pMD2.G, and the expression plasmid of interest using Fugene HD (Promega). 48 h later, viral supernatants were collected and passed through a 0.45 μm filter.

#### Lentiviral transduction

Cells were plated in 6-well dishes at ~30% confluency and transduced with virus 24 h later. 100–150 μL of viral supernatant was added to cells, which were cultured in virus-containing medium for an additional 48 h. Populations of cells co-expressing each construct were isolated using fluorescence-activated cell sorting on a FACSAria Fusion (BD Biosciences) and expanded for subsequent experiments. Bulk-sorted populations were selected for cells expressing the following constructs: ErkKTR-iRFP-2A-H2B-tRFP; ErkKTR-iRFP; ErkKTR-iRFP∷EGFR-FusionRed, ErkKTR-iRFP∷tetON-shYAP; ErkKTR-iRFP∷YFP-YAP^5SA^. Clonal populations were selected for cells expressing the following constructs: ErkKTR-iRFP-2A-H2B-tRFP∷BFP-SSPB-SOScat-2A-PuroR-2A-iLID-CAAX; ErkKTR-iRFP∷Myr-FusionRed-Cry2Drop-EGFR.

#### Time-lapse imaging

Imaging experiments were conducted on a Nikon Eclipse Ti confocal microscope equipped with a Yokogawa CSU-X1 spinning disk, a Prior Proscan III motorized stage, an Agilent MLC 400B laser launch, and a cooled iXon DU897 EMCCD camera. An environmental chamber was used to maintain cells at 37°C and 5% CO_2_ during imaging. In microscopy experiments using optogenetic stimuli, an X-cite XLED1 light source linked to a Polygon400 Mightex Systems digital micromirror device was used to stimulate cells with 500-ms pulses of 450 nm blue light every 1 min, which we define as continuous blue light stimulation. All images were collected using a 20× air, 40× air, or 60× oil objective. Time-lapse images were acquired every 1–3 min.

#### Immunoblotting analysis

For optogenetic experiments, cells were continuously illuminated with 10 s ON/20 s OFF cycles of 450 nm light at 8 V delivered by LEDs on a custom-printed circuit board, placed atop a foil-wrapped box within a tissue culture incubator maintained at 37°C and 5% CO_2_ (Goglia et al., 2020). Cells cultured on polyacrylamide substrata or tissue culture-grade polystyrene (TCPS) in 6-well tissue-culture plates were washed with PBS and lysed with SDS buffer. Cell scrapers were used to remove cells from the surface of each substratum. Cell lysates were then transferred to 1.5 mL Eppendorf tubes, vortexed for 10–15 s, heated at 80°C for 10 min, and centrifuged at 15,000 rpm for 5 min at room temperature. Protein concentrations were measured using the Pierce BCA Protein Assay Kit (Thermo Scientific). Lysates were then mixed with NuPAGE Sample Reducing Agent (Invitrogen) and NuPAGE LDS Sample Buffer (Invitrogen), heated at 80°C for 10 min, and separated by SDS-PAGE. Samples were resolved on SDS-PAGE gels, transferred to nitrocellulose membranes, and blocked with Odyssey Blocking Buffer (LI-COR Biosciences) for 1 h at room temperature before incubating overnight at 4°C in Odyssey Blocking Buffer containing primary antibodies. The following antibodies were used: anti-phospho-p44/42 Erk1/2 (Cell Signaling 9101), anti-p44/42 Erk1/2 (Cell Signaling 4696), anti-phospho-EGFR (Cell Signaling 3777), anti-EGFR (Cell Signaling 2232 or Cell Signaling 4267), anti-YAP/TAZ (Cell Signaling 8418), or anti-GAPDH (Cell Signaling 2118). Membranes were then washed three times for 5 min each with TBST and incubated for 1 h at room temperature in Odyssey Blocking Buffer containing IRDye 680RD goat-anti-mouse and 800CW goat-anti-rabbit fluorescent secondary antibodies (LI-COR Biosciences). Blots were washed three times for 5 min each with TBST and imaged using a LI-COR Odyssey CLx imaging system. Immunoblot images were analyzed using Fiji (Schindelin et al., 2012).

#### Immunofluorescence analysis

Cells were fixed with 4% paraformaldehyde for 15 min, washed three times for 5 min each with PBS, and incubated for 1 h at room temperature in blocking buffer consisting of PBS, 0.3% Triton X-100 (Sigma Aldrich), and 5% normal goat serum (Sigma Aldrich). Samples were then incubated overnight at 4°C in antibody dilution buffer consisting of PBS, 0.3% Triton X-100, and 2% bovine serum albumin (Sigma Aldrich) containing primary antibodies. The following antibodies were used: anti-EGFR (Santa Cruz Biotechnology sc-101), anti-E-cadherin (Cell Signaling 3195 or Thermo Fisher 13-1900), anti-YAP/TAZ (Cell Signaling 8418). The next day, samples were washed three times for 5 min each with PBS and incubated for 1 h at room temperature in antibody dilution buffer containing Alexa Fluor-conjugated secondary antibodies (Invitrogen). Samples were then washed three times for 5 min each with PBS, incubated for 5 min at room temperature in PBS containing Hoechst 33342 (Invitrogen), and washed twice with PBS before imaging under confocal microscopy.

#### Quantitative real-time PCR

qRT-PCR was conducted as described previously (Pang et al., 2016). RNA was extracted using TRIzol and cDNA was synthesized using a Verso cDNA synthesis kit (Thermo Fisher). Transcript levels were measured using a Bio-Rad Mini Opticon instrument and iTaq Universal SYBR Green Supermix (Bio-Rad). The transcript level of EGFR was normalized to that of the 18S ribosomal subunit in the same sample.

#### EGF-488 internalization assay

Cells were seeded on polyacrylamide substrata and swapped to GF-free medium as described above. Cells were then treated with 20 ng/mL EGF-Alexa-488 (EGF-488) (Thermo Fisher), fixed with 4% paraformaldehyde, stained with Hoechst 33342 (Invitrogen), or immunostained for EGFR and E-cadherin. Samples were imaged by confocal microscopy using a 60× oil objective.

#### EGF-488 membrane binding assay

Cells were seeded on polyacrylamide substrata and swapped to GF-free medium as described above. Samples were then placed and kept on ice, and treated with 20 ng/mL EGF-488 10 min later. 10 min after EGF-488 treatment, samples were rinsed once with ice-cold PBS to remove residual EGF-488, and fixed with 4% paraformaldehyde. Parental MCF10A cells in Figures 5F and 5G were immunostained for EGFR and E-cadherin. Samples were imaged using an inverted Nikon Ti-E equipped with a Yokogawa CSU21 spinning disk, Agilent high power MLC400 laser launch, and Hamamatsu sCMOS Fusion BT camera, using a 60× oil objective.

### QUANTIFICATION AND STATISTICAL ANALYSIS

#### KTR image analysis

Multi-time point TIFF stacks of the KTR channel from time-lapse microscopy experiments were imported into Fiji, subtracted of background intensity measured in regions absent of cells, and used to measure Erk dynamics in individual cells. Nuclear and cytoplasmic regions of randomly selected cells were segmented in Fiji, and the mean gray value (intensity) was measured at each time point. The KTR-reported Erk activity (C/N ratio) of a given cell was calculated by dividing the cytoplasmic KTR intensity by the nuclear KTR intensity at each time point.

Subsequent analyses of C/N ratios were conducted in MATLAB. To eliminate internal noise from C/N trajectories, C/N ratios were averaged with the previous and subsequent frames. Pulses of Erk activity, defined as a 20% increase in the C/N ratio relative to neighboring time points, were identified using the peakfinder plugin (https://www.mathworks.com/matlabcentral/fileexchange/25500-peakfinder-x0-sel-thresh-extrema-includeendpoints-interpolate). KTR AUCs for individual cells were calculated by subtracting the C/N ratio before stimulation from C/N ratios at all time points and summing values at each time point over the period of interest. C/N ratios measured at fixed time points (e.g. at 0 min or 120 min after stimulation) were calculated as the mean of time points 10 min before or after the time of interest.

#### Quantifying EGF-488 internalization

Quantification of EGF-488 puncta was performed in MATLAB. First, *z*-stack images of EGF-488-treated samples were subtracted of background intensity by subtracting a gaussian-blurred version of each *z*-slice from its complementary raw image. Background subtracted images were subjected to intensity thresholding to detect EGF-positive pixels, which were then used to detect 3D EGF-positive objects across the *z*-stack image. EGF-positive objects were then subjected to thresholding by size to eliminate pixel noise and objects larger than individual puncta. For samples immunostained for EGFR and E-cadherin, intensity and size thresholding were conducted for both EGFR and EGF-488 puncta, which were filtered for pixels doubly positive for EGFR and EGF-488. For each biological replicate, the total volume of puncta was normalized to the number of nuclei present.

#### Quantifying EGF-488 membrane binding

To quantify EGF-488 puncta localized across the entirety of the cell membrane, 3D *z*-stack images were converted to max intensity projection images to collapse EGF-488 signals onto a single *xy* plane. These max intensity projections are particularly important for rounded tissues on 0.1 kPa substrata, where the entire apical surface of a cell was not captured in a single *z*-slice, making alternative approaches (e.g., measuring the total EGF fluorescence intensity across the membrane) extremely challenging to implement. Using the E-cadherin channel, cells were then segmented using CellPose (Stringer et al., 2021). EGF-488 images were subjected to a rolling ball background subtraction and gaussian filter in Fiji, and the area of EGF-488 puncta per cell was quantified in MATLAB. The EGF-488 channel was subjected to size and intensity thresholding to detect EGF-positive objects. For each cell segmented in CellPose, the total area of EGF-positive objects was quantified.

#### Statistical analysis and replicates

All statistical analyses were performed in GraphPad Prism and are described in each figure legend where statistical comparisons were performed. Paired *t* tests were performed for immunoblotting results, in which results were analyzed relative to one normalized condition within the experiment. Unpaired *t* tests were performed for all other pairwise comparisons unless three or more groups were compared, in which case a one-way ANOVA and Tukey *post hoc* test was performed. Except for experiments in Figure 2, experiments were conducted with at least 3 biological replicates, defined as biologically distinct samples aimed to capture biological variation. *n* was defined as either the number of biological replicates or the number of cells analyzed from a reported number of biological replicates and is reported in each figure legend.

## Supplementary Material

1

2

3

4

5

6

7

## Figures and Tables

**Table:** KEY RESOURCES TABLE

REAGENT or RESOURCE	SOURCE	IDENTIFIER
Antibodies		
Phospho Erk 1/2 rabbit antibody	Cell Signaling Technologies	Cat #9101
		RRID: AB_331646
Phospho Erk 1/2 mouse antibody	Cell Signaling Technologies	Cat # 4696
		RRID: AB_390780
Phospho Y1068 EGFR rabbit antibody	Cell Signaling Technologies	Cat # 3777
		RRID: AB_2096270
EGFR rabbit antibody	Cell Signaling Technologies	Cat # 2232
		RRID: AB_331707
EGFR rabbit antibody	Cell Signaling Technologies	Cat # 4267
		RRID: AB_2246311
EGFR mouse antibody	Santa Cruz Biotechnology	Cat # sc-101
		RRID: AB_627494
YAP/TAZ rabbit antibody	Cell Signaling Technologies	Cat #8418
		RRID: AB_10950494
GAPDH rabbit antibody	Cell Signaling Technologies	Cat #2118
		RRID: AB_561053
IRDye 680RD Goat anti-Mouse IgG antibody	LI-COR Biosciences	Cat # 926-68070
		RRID: AB_10956588
IRDye 800CW Goat anti-Rabbit IgG antibody	LI-COR Biosciences	Cat #926-32211
		RRID: AB_621843
E-cadherin rabbit antibody	Cell Signaling Technologies	Cat #3195
		RRID: AB_2291471
E-cadherin rat antibody	Thermo Fisher Scientific	Cat # 13-1900
		RRID: AB_2533005
Bacterial and virus strains		
Stellar Chemically Competent Cells	ClonTech Laboratories	Cat # 636763
Chemicals, peptides, and recombinant proteins		
DMEM/F-12	Gibco	Cat # 11320033
Horse serum	Gibco	Cat # 16050122
Epidermal growth factor (EGF)	R&D Systems	Cat # 236-EG
Hydrocortisone	Sigma Aldrich	Cat# H0888
Cholera toxin	Sigma Aldrich	Cat # C8052
Insulin	Sigma Aldrich	Cat # I6634
Penicillin/Streptomycin/Glutamine	Gibco	Cat # 10378016
Bovine serum albumin	Sigma Aldrich	Cat # A7906
Fibronectin	Corning	Cat # CB-40008A
U0126	Cell Signaling Technologies	Cat # 9903
Gefitinib	Cell Signaling Technologies	Cat # 4765
Doxycycline	Fisher Scientific	Cat # NC0424034
DMSO	Sigma Aldrich	Cat # D8418
ClonAmp HiFi PCR polymerase	ClonTech Laboratories	Cat # 639298
PrimeSTAR GXL DNA Polymerase	ClonTech Laboratories	Cat # R050B
inFusion HD cloning kit	ClonTech Laboratories	Cat # 638911
DMEM/F12 (3:1) without calcium	Life Technologies	Cat #90-5010
Sodium bicarbonate	Sigma Aldrich	Cat # S5761
Transferrin	Sigma Aldrich	Cat # T2252
T3 (3,3’,5-triiodo-L-thyronine)	Sigma Aldrich	Cat # T2877
EGF, Alexa Fluor 488-conjugated	Invitrogen	Cat # E13345
Aminopropyltrimethoxysilane	Sigma Aldrich	Cat # 281778
Glutaraldehyde	Sigma Aldrich	Cat # 340855
40% acrylamide solution	Bio-Rad	Cat # 1610140
2% bis-acrylamide solution	Bio-Rad	Cat # 161-0142
Sulfo-SANPAH	Thermo Fisher Scientific	Cat # 22589
N,N,N′,N′-Tetramethylethylenediamine (TEMED)	Sigma Aldrich	Cat # T9281
Ammonium persulfate (APS)	Sigma Aldrich	Cat # A3678
Fugene HD	Promega	Cat # E2311
Verso cDNA synthesis kit	Thermo Fisher Scientific	Cat # AB1453A
iTaq Universal SYBR Green Supermix	Bio-Rad	Cat # 1725120
Experimental models: Cell lines		
MCF10A human mammary epithelial cells, clone 5E	(Janes et al., 2010)	RRID: CVCL_0598
ErkKTR-iRFP-2A-H2B-tRFP (MCF10A, clone 5E)	This paper	N/A
ErkKTR-iRFP (MCF10A, clone 5E)	This paper	N/A
ErkKTR-iRFP-2A-H2B-tRFP∷BFP-SSPB-SOScat-2A-PuroR-2A-iLID-CAAX (MCF10A, clone 5E)	This paper	N/A
ErkKTR-iRFP∷Myr-FusionRed-Cry2Drop-EGFR (MCF10A, clone 5E)	This paper	N/A
ErkKTR-iRFP∷TetON-shYAP (MCF10A, clone 5E)	This paper	N/A
ErkKTR-iRFP∷YFP-YAP5SA (MCF10A, clone 5E)	This paper	N/A
ErkKTR-iRFP∷EGFR-FusionRed (MCF10A, clone 5E)	This paper	N/A
ErkKTR-iRFP∷H2B-RFP (CD-1 mouse primary keratinocytes)	This paper	N/A
Lenti-X HEK 293T cells	ClonTech Laboratories	Cat # 632180
Oligonucleotides		
Human EGFR forward qRT-PCR primer: 5’ - CGTGGCAAGTCCCCCAGTGA - 3’	(Guturi et al., 2012)	N/A
Human EGFR reverse qRT-PCR primer: 5’ - GCAGACCAGGCAGTCGCTCTC- 3’	(Guturi et al., 2012)	N/A
Human 18S rRNA forward qRT-PCR primer: 5’ - CGGCGACGACCCATTCGAAC - 3’	(Rabie etal., 2021)	N/A
Human 18S rRNA reverse qRT-PCR primer: 5’ - GAATCGAACCCTGATTCCCCGTC - 3’	(Rabie etal., 2021)	N/A
Recombinant DNA		
pHR ErkKTR-iRFP-2A-H2B-tRFP	(Goglia et al., 2020)	N/A
pHR BFP-SSPB-SOScat-2A-PuroR-2A-iLID-CAAX	(Goglia et al., 2020)	N/A
pHR ErkKTR-iRFP	(Dine et al., 2018)	Addgene # 111510
pHR EGFR-FusionRed	This paper	Addgene # 179263
pHR Myr-FusionRed-Cry2Drop-EGFR	This paper	Addgene # 179262
pCMV-dR8.91 lentivirus packaging plasmid	Gift from Prof. DidierTrono, EPFL	Addgene #12263
pMD2.G lenti helper plasmid	Gift from Prof. DidierTrono, EPFL	Addgene # 12259
TetON-shYAP	Gift from Joan Massague, MSKCC	Addgene # 115667
pHR YFP-YAP5SA	Gift from Erik Sahai; cloned into pHR vector	Addgene # 112285
Software and algorithms		
MATLAB R2021a	MathWorks	RRID: SCR_001622
Peakfinder plugin	Nathanael Yoder	https://www.mathworks.com/matlabcentral/fileexchange/25500-peakfinder-x0-sel-thresh-extrema-includeendpoints-interpolate
Fiji	(Schindelin et al., 2012)	http://fiji.sc; RRID: SCR_00228
CellPose	(Stringer et al., 2021)	https://github.com/MouseLand/cellpose
GraphPad Prism v5.0	GraphPad	https://www.graphpad.com/
MATLAB analysis code	This paper	https://doi.org/10.5281/zenodo.5735648

## Highlights

- Soft substrata decrease the amplitude and frequency of Erk dynamics
- The intracellular EGFR/Erk pathway remains signaling competent on soft substrata
- Soft substrata decrease the efficiency of EGFR activation and EGF membrane binding
- Overexpressing EGFR is sufficient to increase Erk signaling on soft substrata
