# Supplementary material for: Substratum stiffness regulates Erk signaling dynamics through receptor-level control
Source: Cell Rep. Author manuscript; Available in PMC 2022 Jan 13. (PMC8756379; doi:10.1016/j.celrep.2021.110181)
Supplement: 1 [file NIHMS1767658-supplement-1.pdf]

Cell Reports, Volume *37*

Supplemental information

Substratum stiffness regulates Erk signaling dynamics through receptor-level control

Payam E. Farahani, Sandra B. Lemke, Elliot Dine, Giselle Uribe, Jared E. Toettcher, and Celeste M. Nelson

Supplementary Figures

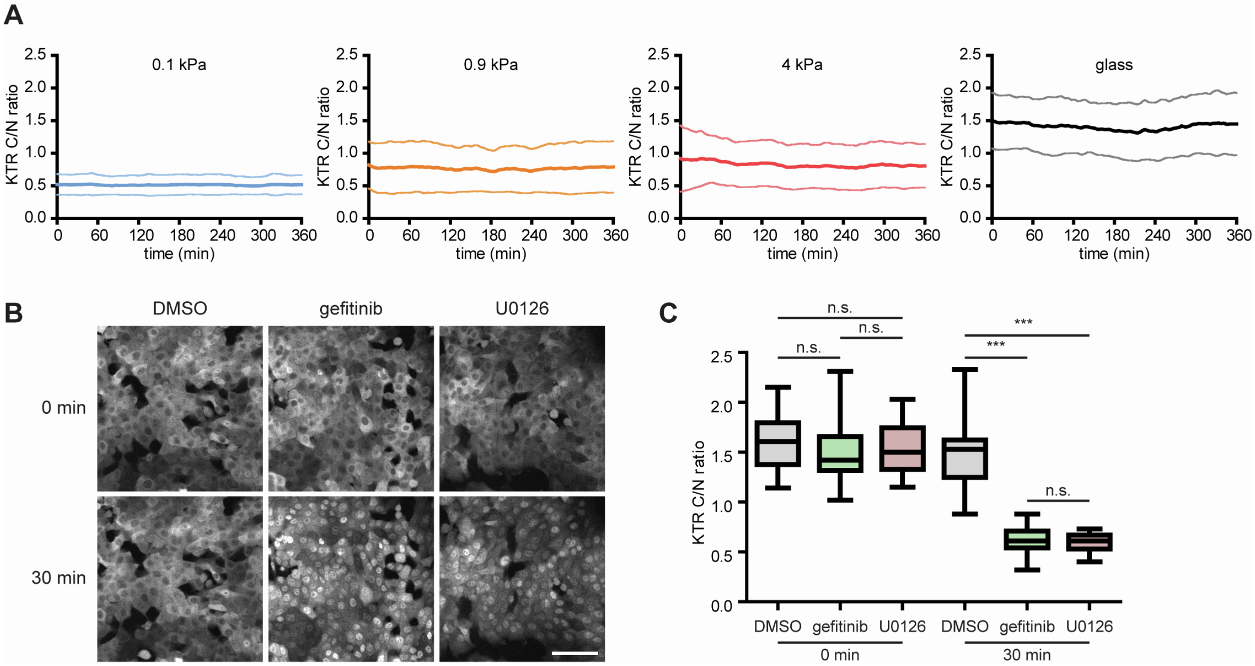

Figure S1. Steady-state signaling in growth medium requires signaling through EGFR. (**A**) Mean ± S.D. population-averaged KTR C/N ratios measured in Figure 1 confirm that Erk signaling is at steady-state levels over the duration of time-lapse imaging. (**B**) Representative images of MCF10A cells in growth medium treated with DMSO, gefitinib (5 µM), or U0126 (10 µM) (scale bar, 100 µm). (**C**) C/N ratios before and 30 min after drug treatment. Boxes and whiskers represent the 25-75th percentiles and minima and maxima, respectively. Mean values are indicated by horizontal lines. ***, $p < 0.001$; n.s., not significant using one-way ANOVA and Tukey *post hoc* tests. For each condition, $n = 30$ cells from 3 biological replicates. Related to Figure 1.

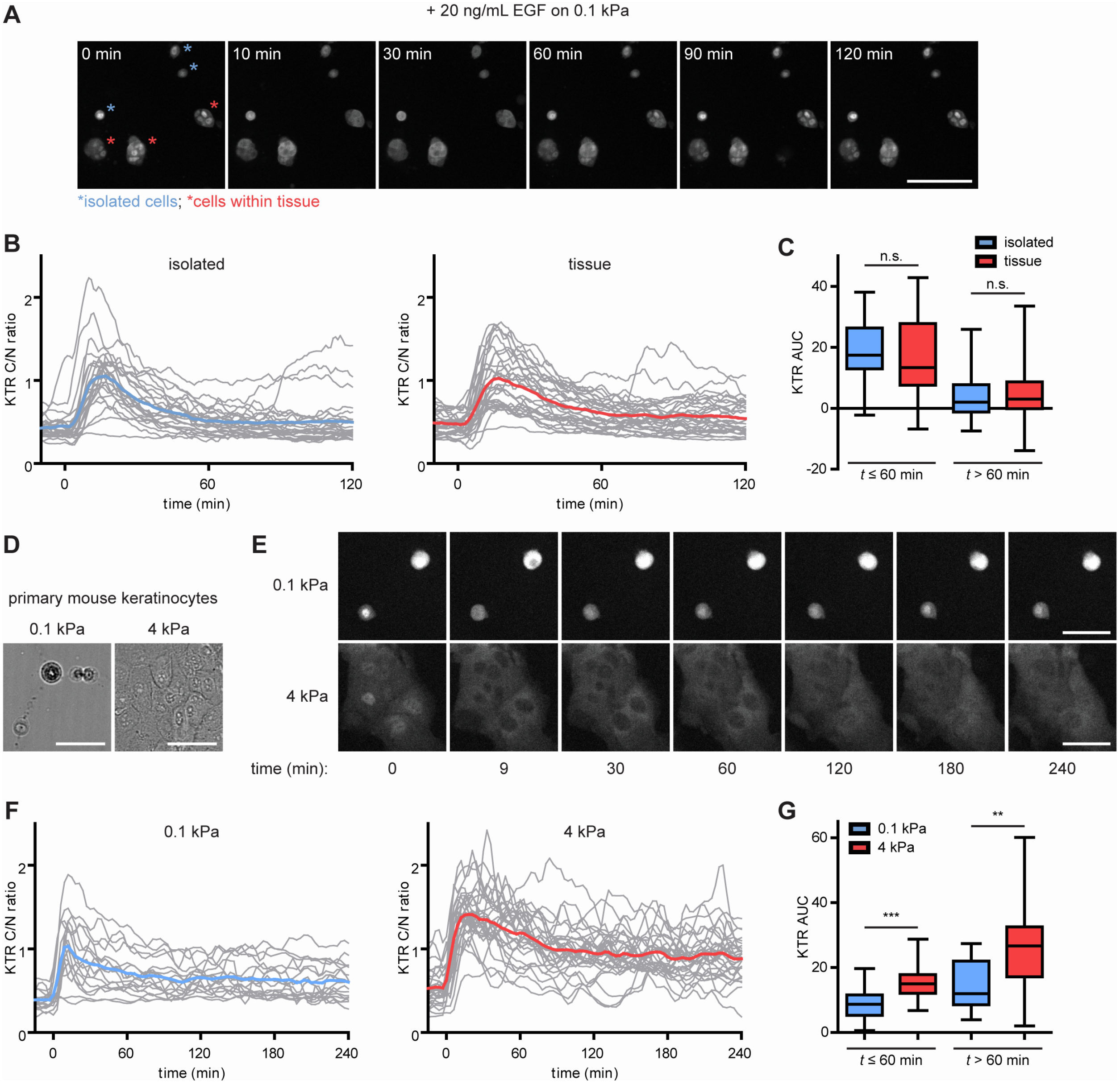

Figure S2. Extended analysis of growth factor-stimulated Erk signaling. (**A**) Representative time-lapse images of MCF10A cells cultured on soft substrata and treated with EGF (20 ng/mL). Isolated cells and cells within tissues are denoted by blue and red asterisks, respectively (scale bar, 100 µm). (**B**) Mean Erk trajectories of MCF10A cells after EGF treatment, with the responses of individual cells represented by gray lines. (**C**) Cumulative Erk responses at early and late time points as measured by the KTR AUC. Boxes and whiskers represent the 25-75th percentiles and minima and maxima, respectively. Mean values are indicated by horizontal lines. Figures S2B,C: for each condition, *n* > 20 cells from 3 biological replicates. (**D**) Representative images of keratinocytes cultured on soft or stiff substrata (scale bars, 50 µm). (**E**) Representative time-lapse images of keratinocytes on soft or stiff substrata treated with EGF (20 ng/mL) (scale bars, 50 µm). (**F**) Mean Erk trajectories of keratinocytes after EGF treatment, with the responses of individual cells represented by gray lines. (**G**) Cumulative Erk responses at early and late time points as measured by the KTR AUC. Boxes and whiskers represent the 25-75th percentiles and

minima and maxima, respectively. Mean values are indicated by horizontal lines. Figures S2F,G: for each condition, $n > 15$ cells from 3 biological replicates. ***, $p < 0.001$; **, $p < 0.01$; n.s., not significant using an unpaired *t*-test. Related to Figure 2.

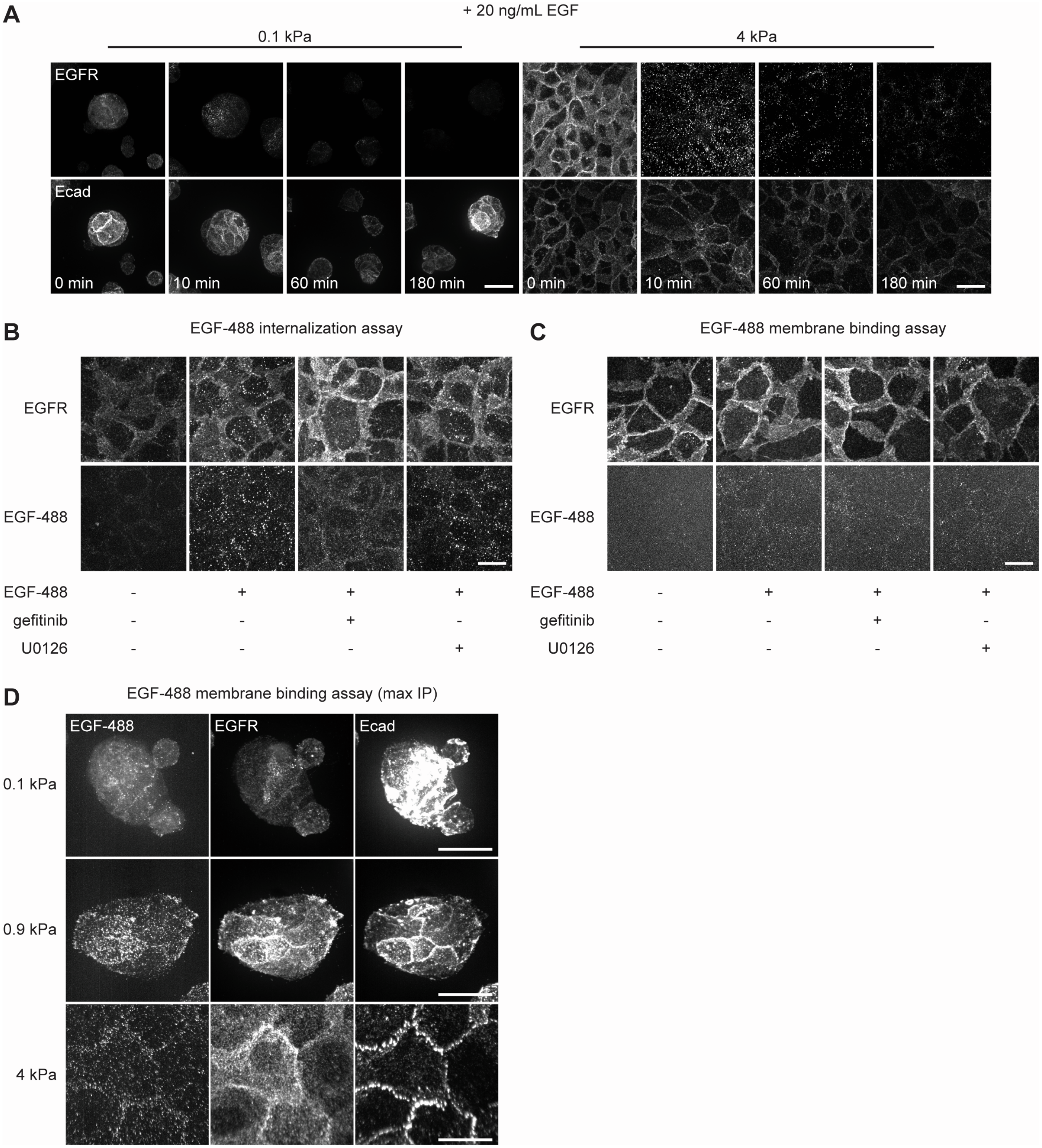

Figure S3. Extended analyses of EGFR internalization, ligand internalization, and ligand membrane binding. (**A**) Max IP images of MCF10A cells cultured on soft or stiff substrata in GF-free medium, stimulated with EGF (20 ng/mL), and subjected to immunostaining analysis for EGFR and E-cadherin (Ecad) (scale bars, 30 µm). Representative max IP images of cells subjected to (**B**) EGF-488 internalization and (**C**) EGF-488 membrane binding assays after pre-treatment with gefitinib (5 µM) or U0126 (10 µM) (scale bars, 20 µm). (**D**) Max IP images of cells on different substrata subjected to EGF-488 membrane binding assays and immunostaining for EGFR and Ecad, from Figure 5F (scale bars, 20 µm). Related to Figure 5.

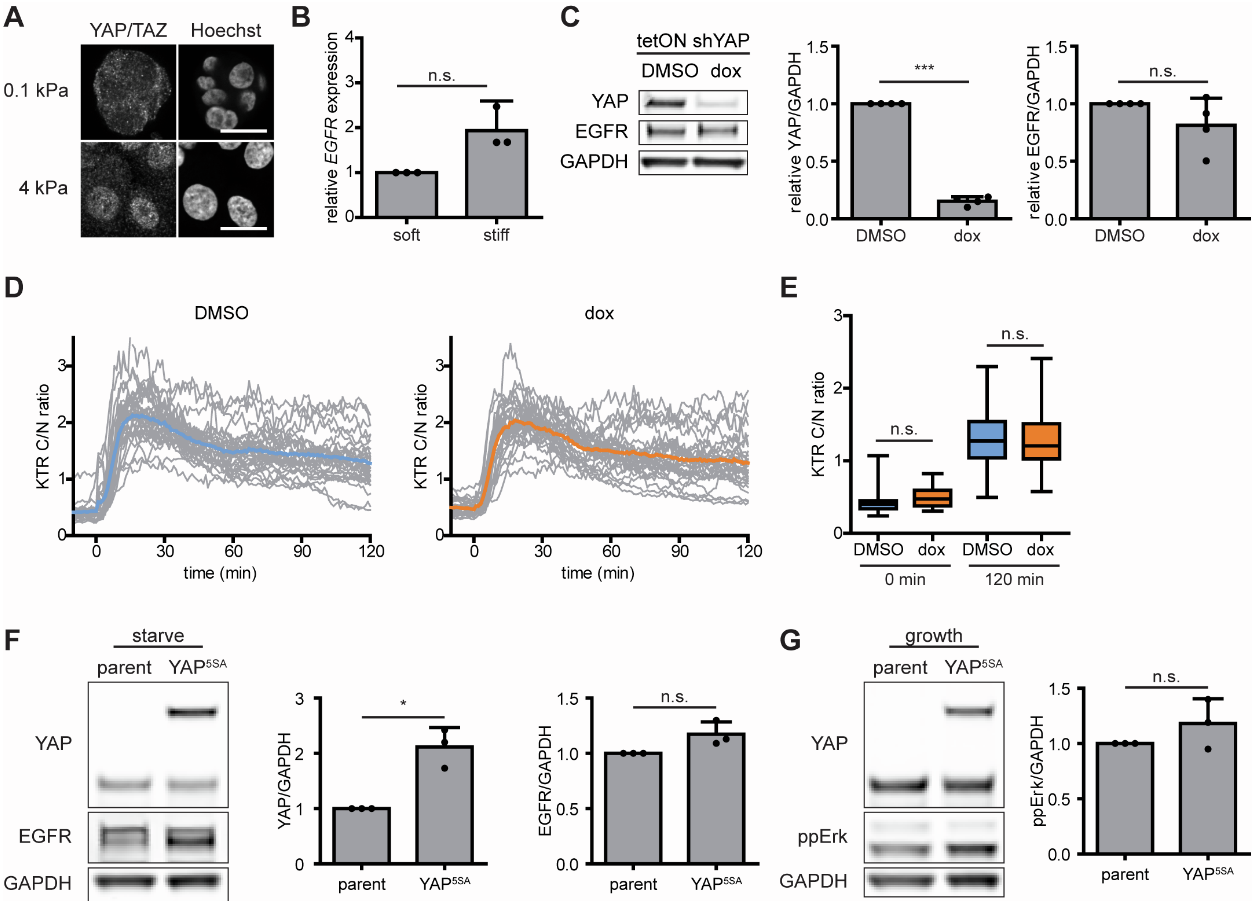

Figure S4. Substratum stiffness modulates EGFR/Erk signal transmission independently of YAP. (**A**) Representative images of MCF10A cells cultured in growth medium on soft or stiff substrata and subjected to immunofluorescence analysis for YAP/TAZ (scale bars, 20 µm). (**B**) Mean + S.D. transcript levels of *EGFR* in cells cultured in GF-free medium on soft or stiff substrata. $n = 3$ biological replicates. (**C**) Depleting YAP expression by inducing YAP-targeting shRNA (shYAP) with doxycycline (dox) decreases the levels of YAP but not EGFR in cells cultured on plastic. $n = 4$ biological replicates. (**D**) Mean Erk trajectories of shYAP-expressing cells and control cells after EGF treatment (20 ng/mL), with the responses of individual cells represented by gray lines. (**E**) C/N ratios before and 120 min after EGF treatment. Boxes and whiskers in (E) represent the 25-75th percentiles and minima and maxima, respectively. For each condition, $n = 30$ cells from 3 biological replicates. n.s., not significant using an unpaired *t*-test. (**F**) Cells cultured in GF-free medium on soft substrata expressing a constitutively active YAP (YFP-YAP^{5SA}) have higher levels of YAP expression and comparable levels of EGFR. $n = 3$ biological replicates. (**G**) YFP-YAP^{5SA}-expressing and control cells exhibit similar levels of ppErk when cultured in growth medium on soft substrata. $n = 3$ biological replicates. Figures S4B-C,F-G: n.s., not significant; *, $p < 0.05$; ***, $p < 0.001$ using a paired *t*-test. Error bars denote S.D. Related to Figure 5.

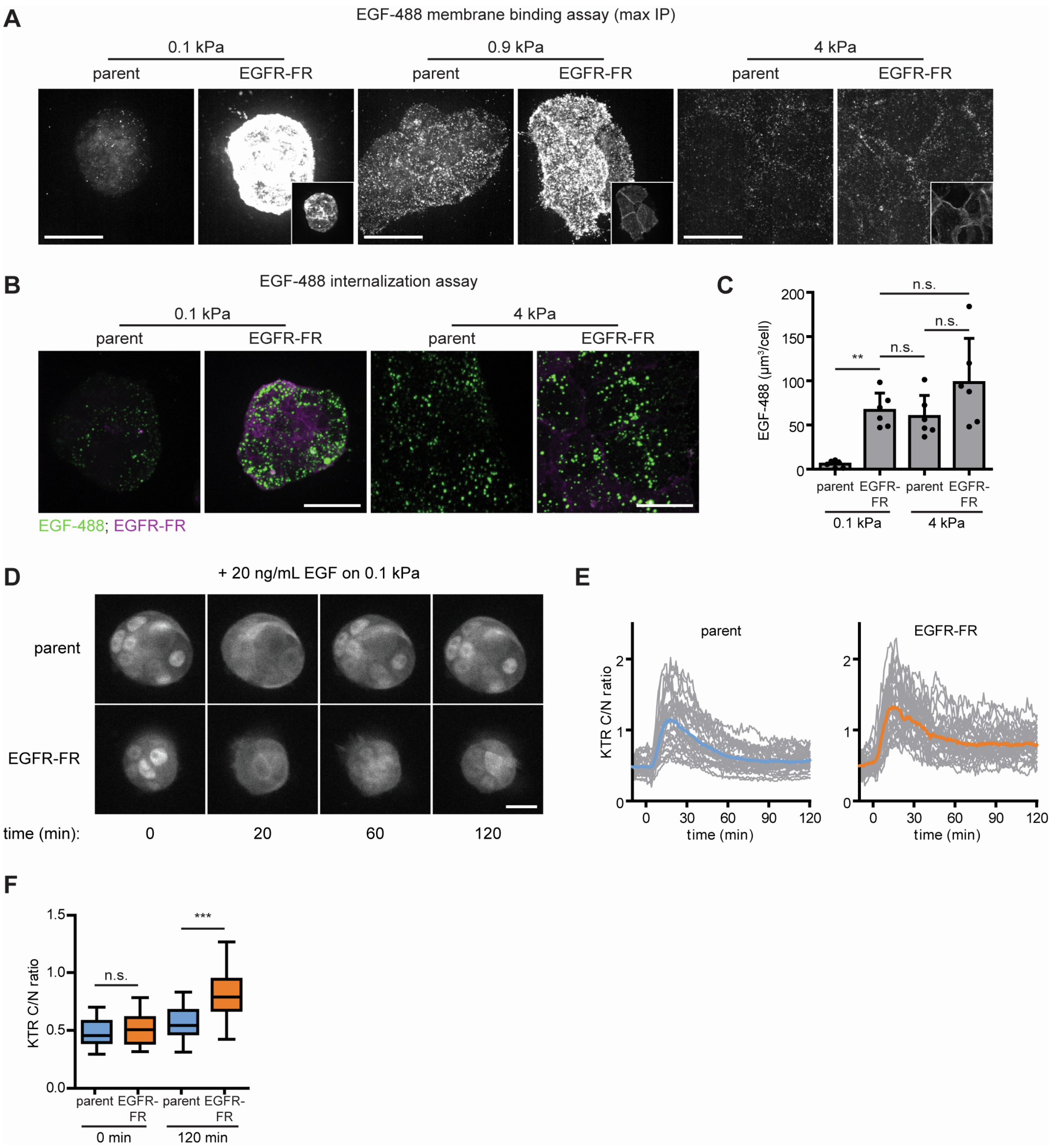

Figure S5. EGF internalization and downstream Erk signaling under EGFR overexpression. (**A**) Max IP EGF-488 images of EGFR-FR-expressing cells or parental cells subjected to EGF-488 membrane binding assays, from Figure 6B. Inset images display EGFR-FR in EGFR-FR-expressing cells (scale bars, 20 µm). (**B**) Representative max IP images of parental or EGFR-FR-expressing cells on soft or stiff substrata treated with EGF-488 (20 ng/mL) for 20 min (scale bars, 20 µm). (**C**) Mean + S.D. quantification from (A). $n = 6$ biological replicates. n.s., not significant; **, $p < 0.01$ using one-way ANOVA and Tukey *post hoc* tests. (**D**) Representative time-lapse images of parental and EGFR-FR-expressing cells on soft

substrata stimulated with EGF (20 ng/mL) (scale bar, 20 μm). (**E**) Mean Erk trajectories of parental and EGFR-FR-expressing cells on soft substrata after 20 ng/mL EGF treatment, with the responses of individual cells represented by gray lines. (**F**) C/N ratios before and 120 min after EGF treatment. Boxes and whiskers represent the 25-75th percentiles and minima and maxima, respectively. Mean values are indicated by horizontal lines. Figures S5D,E: for each condition, $n = 30$ cells from 3 biological replicates. n.s., not significant; ***, $p < 0.001$ using an unpaired *t*-test. Related to Figure 6.
